# Genetic variants in pachyonychia congenita-associated keratins increase susceptibility to tooth decay

**DOI:** 10.1371/journal.pgen.1007168

**Published:** 2018-01-22

**Authors:** Olivier Duverger, Jenna C. Carlson, Chelsea M. Karacz, Mary E. Schwartz, Michael A. Cross, Mary L. Marazita, John R. Shaffer, Maria I. Morasso

**Affiliations:** 1 Laboratory of Skin Biology, National Institute of Arthritis and Musculoskeletal and Skin Diseases, National Institutes of Health, Bethesda, MD, United States of America; 2 Department of Human Genetics, University of Pittsburgh, Pittsburgh, PA, United States of America; 3 Department of Biostatistics, University of Pittsburgh, Pittsburgh, PA, United States of America; 4 Pachyonychia Congenita Project, Holladay, UT, United States of America; 5 Center for Craniofacial and Dental Genetics, University of Pittsburgh, Pittsburgh, PA, United States of America; 6 Department of Oral Biology, School of Dental Medicine, Clinical and Translational Science Institute, Department of Psychiatry, School of Medicine, University of Pittsburgh, Pittsburgh, PA, United States of America; Stanford University School of Medicine, UNITED STATES

## Abstract

Pachyonychia congenita (PC) is a cutaneous disorder primarily characterized by nail dystrophy and painful palmoplantar keratoderma. PC is caused by mutations in *KRT6A*, *KRT6B*, *KRT6C*, *KRT16*, and *KRT17*, a set of keratin genes expressed in the nail bed, palmoplantar epidermis, oral mucosal epithelium, hair follicle and sweat gland. RNA-seq analysis revealed that all PC-associated keratins (except for *Krt6c* that does exist in the mouse genome) are expressed in the mouse enamel organ. We further demonstrated that these keratins are produced by ameloblasts and are incorporated into mature human enamel. Using genetic and intraoral examination data from 573 adults and 449 children, we identified several missense polymorphisms in *KRT6A*, *KRT6B* and *KRT6C* that lead to a higher risk for dental caries. Structural analysis of teeth from a PC patient carrying a p.Asn171Lys substitution in keratin-6a (K6a) revealed disruption of enamel rod sheaths resulting in altered rod shape and distribution. Finally, this PC-associated substitution as well as more frequent caries-associated SNPs, found in two of the *KRT6* genes, that result in p.Ser143Asn substitution (rs28538343 in *KRT6B* and rs151117600 in *KRT6C*), alter the assembly of K6 filaments in ameloblast-like cells. These results identify a new set of keratins involved in tooth enamel formation, distinguish novel susceptibility loci for tooth decay and reveal additional clinical features of pachyonychia congenita.

## Author summary

Tooth decay, more commonly known as dental cavities, is the most common chronic disease worldwide, both in children and in adults. It consists in the destruction of tooth enamel, the outer layer of the teeth, by acid-producing bacteria. Enamel is the hardest tissue in the body, comprised of 96% minerals. However, it contains a small fraction of proteins that is important for its resistance to mechanical stress and decay. Here we show that this protein fraction contains a set of structural proteins (K6a, K6b, K6c, K16 and K17) that belong to the keratin family and are present specifically in the skin of the palms and soles, as well as in nails. We further show that common genetic mutations that affect the composition of these proteins lead to an increased number of cavities. Rare mutations in these keratins lead to a human disease called pachyonychia congenita (PC) and characterized by severe nail malformations and lesions in the skin of the palms and soles. Analysis of wisdom teeth from one of these patients showed that their enamel exhibited structural defects. These results demonstrate that these keratins are important components of tooth enamel and that common genetic variants in the genes that encode them influence tooth decay risk in the general population.

## Introduction

Tooth enamel is made of 96% hydroxyapatite minerals, which makes it the hardest tissue in the human body. Enamel is also the first compartment of the tooth to be attacked by dental caries, a chronic disease that affects 42% of children and 92% of adults, with various degrees of severity (number of teeth and tooth surfaces affected) in the general population. Dental caries is initiated at the surface of the tooth by bacteria metabolizing food residues and releasing acids that dissolve enamel minerals [1]. Even though dental caries is influenced by environmental and behavioral factors, there is clear evidence that susceptibility to caries is also driven by host genetic factors [1–3], and genome-wide association studies (GWASs) have revealed genetic variants associated with increased susceptibility to tooth decay [4–8]. These genetic factors may influence the quality of dental tissues and ability to resist carious attacks, may impact other aspects of the oral environment such as the quality of the saliva, enamel pellicle and oral microbiome, and may differ between the primary and permanent dentitions [9].

Tooth enamel is produced in two phases [10]: first, a secretion phase during which enamel matrix proteins are secreted and deposited in a highly structured manner to form enamel rods; and second, a maturation phase during which most enamel matrix proteins are degraded to make space for the full expansion of hydroxyapatite minerals. After maturation, the enamel is left with only 1% of proteins that are abundant near the dentin-enamel junction (DEJ) and expand throughout the enamel as thin layers of enamel rod sheaths located at the interrod region [11]. This organic material has been shown to play a crucial role in the biomechanical properties of enamel [12, 13] and in the resistance to caries [14, 15]. Until recently, the exact composition of the insoluble fraction in this organic material had been uncharacterized, even though there was strong evidence that the highly crosslinked proteins present in mature enamel had biochemical properties similar to those of keratins [11, 16–18]. We showed previously that the organic material in mature enamel is in part composed of epithelial hair keratins, and that missense mutations in *KRT75*, previously linked to common hair disorders, were associated with increased susceptibility to dental caries [19].

In the present study, we focus on the presence in enamel of another set of keratins encoded by genes mutated in pachyonychia congenita (PC), a cutaneous disorder characterized by nail dystrophy and painful palmoplantar keratoderma [20, 21]. Additional features of this disease may include oral leukokeratosis, follicular keratosis, cysts, hyperhidrosis, and natal teeth. Some of these phenotypic traits are consistent with the expression pattern of the keratins involved. Herein we present novel findings that relate this set of keratins to the development of tooth enamel and to the susceptibility to tooth decay.

## Results

### Pachyonychia congenita-associated keratins are produced by ameloblasts and incorporated into mature tooth enamel

**Fig 1:**
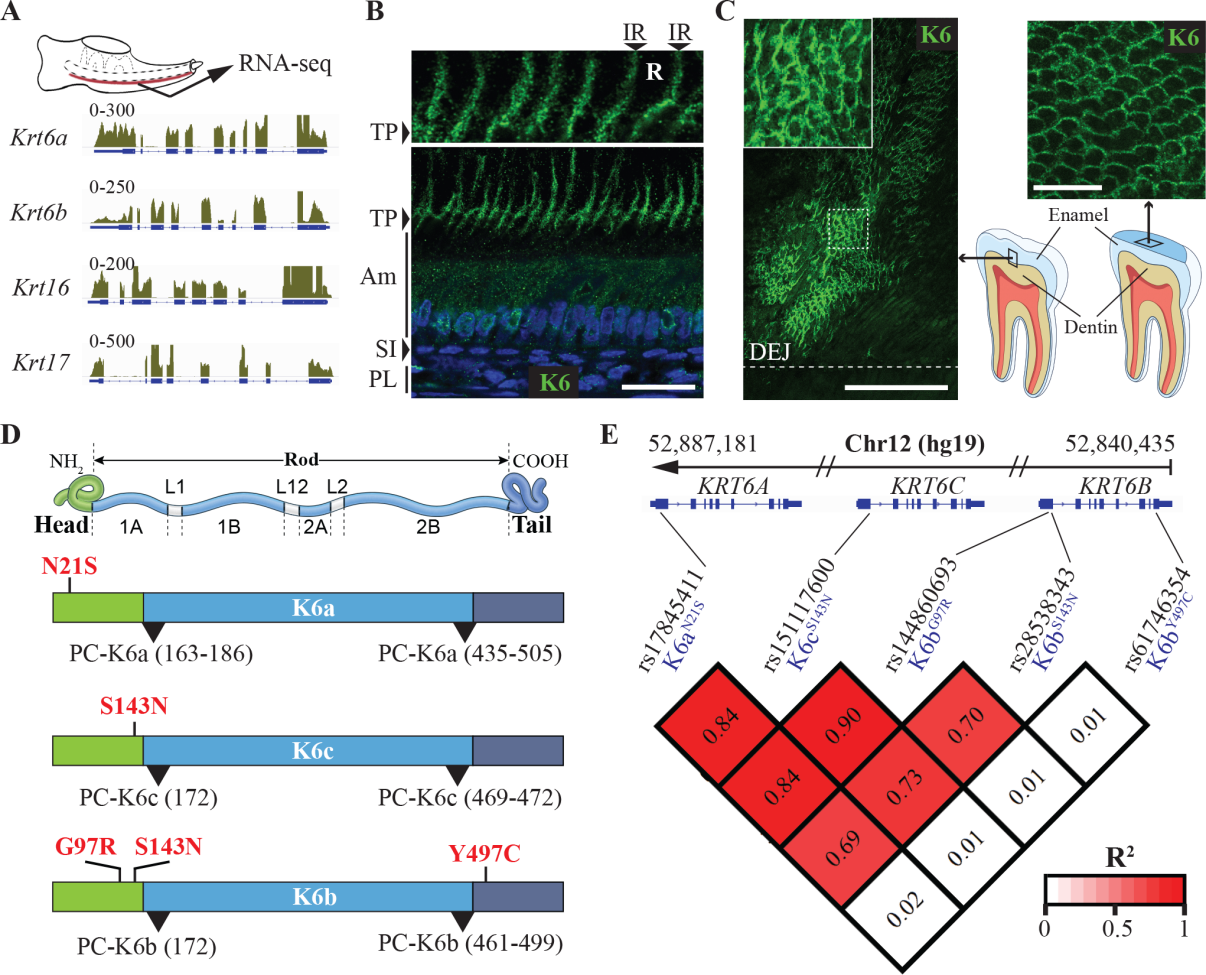
K6 proteins in enamel and genetic association between missense SNPs in *KRT6* genes and tooth decay experience. (**A**) RNA-seq data from mouse enamel organ showing expression of *Krt6a*, *Krt6b*, *Krt16* and *Krt17* in the tissue. The schematics at the top shows a mouse mandible at postnatal day 10 from which the enamel organ (red) was micro-dissected for RNA extraction. Tracks represent the alignment of the RNA-seq reads to the mouse genome for the *Krt6a*, *Krt6b*, *Krt16* and *Krt17* loci. (**B**) Immunohistochemical analysis of K6 distribution in rat enamel organ at postnatal day 10. Anti-K6 antibody recognizes all members of the K6 family (K6a and K6b in rodents). Nuclei are stained with DAPI (blue). Inset at the top shows enlarged view of Tomes’ processes. PL, papillary layer; SI, stratum intermedium; Am, ameloblasts; TP, Tomes’ processes; R, rod; IR, interrod. Scale bar: 50 μm. (**C**) Immunohistochemical detection of K6 on polished sections of human third molars. Antibody raised in guinea pig against the N-terminal of K6 (recognizes K6a, K6b and K6c in humans) was used (green). As indicated by the schematics on the bottom right corner, the image in the left panel was acquired from mesial-distal sections of human third molars and shows the area of enamel adjacent to dentin, whereas the image in the right panel was acquired from transverse section of the crown near the cusps. DEJ, dentin-enamel junction. Scale bars: 100 μm for left panel, 20 μm for right panel. (**D**) Schematics showing the structure of keratin proteins with a central rod domain flanked by a head domain and a tail domain. Shown in red are the position of the five missense SNPs in *KRT6A*, *KRT6B* and *KRT6C* that were found to have significant genetic association with tooth decay risk as measured by three indices of dental caries experience. All SNPs associated with high caries experience in adults resulted in substitutions in the head domain of K6 proteins, while the unique SNP that showed association with tooth decay risk in children only results in a substitution in the tail domain of K6b (Y497C). Areas where mutations leading to PC have so far been identified are highlighted by black arrowheads and are exclusively at the beginning and at the end of the rod domain. (**E**) Linkage disequilibrium data measured by R^2^ between the five missense polymorphisms highlighted in D.

In a previous study, we determined through RNA-seq analysis that subsets of epithelial keratins were expressed in the enamel organ in mouse [19]. Of particular interest was the expression of *Krt6a*, *Krt6b*, *Krt16* and *Krt17* (**Fig 1A**), a set of keratin genes encoding keratin-6a (K6a), keratin-6b (K6b), keratin-16 (K16) and keratin-17 (K17), respectively, and in which mutations in humans lead to pachyonychia congenita (PC-K6a, OMIM #615726; PC-K6b, OMIM #615728; PC-K16, OMIM #167200; PC-K17, OMIM #167210), characterized by nail dystrophy and painful palmoplantar keratoderma [20, 21]. In humans, the *KRT6* family includes a third member (*KRT6C*, encoding K6c), mutations in which have been associated with a milder form of PC with no/minor nail defects (PC-K6c) that was initially reported as palmoplantar keratoderma, non-epidermolytic, focal or diffuse (PPKNEFD, OMIM #615735).

Immunohistochemical analysis revealed that K6 (using an antibody recognizing all K6 proteins) and K17 are produced by rodent ameloblasts but exhibit very distinct distributions (**Fig 1B and S1A Fig**). K6 distribution is relatively diffuse in secretory ameloblasts (**Fig 1B**) while K17 forms characteristic keratin filaments that run throughout the ameloblasts and underlying tissues (stratum intermedium and papillary layer) (**S1A Fig**). At the apex of the ameloblasts and outside the Tomes’ processes, highly specialized structures where the deposition of enamel is coordinated, K6 was detected primarily at the interrod region (**Fig 1B, magnification top panel**). In the same area, K17 staining resulted in parallel transverse bands within the rods in addition to a longitudinal interrod distribution similar to that obtained with K6 staining (**S1A Fig, magnification top panel**). These distributions indicate that K6 and K17 are both incorporated into the enamel matrix but with distinct patterns. To determine whether these keratins were part of the organic material present in mature human enamel, we performed immunohistochemical staining on polished sections of human third molars (**Fig 1C and S1B Fig**). Consistent with its distribution near the apex of rodent ameloblasts, K6 was detected primarily where the enamel rod sheaths are located, at the periphery of the enamel rods (**Fig 1C**). More intense staining was detected at regular intervals near the DEJ, along structures that are likely to correspond to enamel tufts, areas of higher accumulation of organic material (**Fig 1C, left panel**). In addition to intense staining near the DEJ, K6 was detected throughout the thickness of enamel and restricted to the periphery of the rods (**Fig 1C, right panel**). K16 and K17 were detected near the DEJ where they were not restricted to the interrod regions but also present in the core of the rods (**S1B Fig**), a pattern consistent with the distribution of K17 near the apex of rodent ameloblasts. The restricted pattern of K6 distribution at the enamel rod sheaths was confirmed with two different antibodies, a polyclonal antibody raised in guinea-pig against the C-terminus of the protein and a monoclonal antibody raised in mouse against the N-terminus (**S2 Fig**). These results indicate that PC-associated keratins are part of the organic material present in mature enamel but exhibit distinct distributions.

### Several missense SNPs in *KRT6* genes are associated with susceptibility to dental caries in a dentition-dependent manner (primary vs. permanent)

In order to determine if the presence of K6a, K6b, K6c, K16 and K17 in mature human enamel had an impact on the susceptibility to tooth decay, we tested the association between SNPs in the genes encoding these keratins and three measures of dental caries experience assessed in the primary dentition of 449 children (mixed European descent, 6–12 years) and permanent dentition of 573 adults (mixed European descent, 25–50 years). We focused our attention to common missense SNPs that occur at a sufficient frequency (minor allele frequency > 1%) allowing for statistical testing in our unselected population-based cohort. Three missense SNPs in *KRT6A*, eight in *KRT6C*, and seven in *KRT6B* responded to these criteria (**Table 1**). Across all 18 missense SNPs, seven SNPs showed nominal evidence of association (p < 0.05) with at least one measure of dental caries experience in either adults or children, and the following five SNPs exhibited associations with all three measures of dental caries experience (**Table 1**):

- *KRT6A*:*c*.*61A>G* (rs17845411) leading to p.Asn21Ser (K6a^N21S^).
- *KRT6C*:*c*.*428G>A* (rs151117600) leading to p.Ser143Asn (K6c^S143N^).
- *KRT6B*:*c*.*289G>A* (rs144860693) leading to p.Gly97Arg (K6b^G97R^).
- *KRT6B*:*c*.*428G>A* (rs28538343) leading to p.Ser143Asn (K6b^S143N^).
- *KRT6B*:*c*.*1490A>G* (rs61746354) leading to p.Tyr497Cys (K6b^Y497C^).

**Table 1:** Genetic association of missense SNPs in *KRT6A*, *KRT6B* and *KRT6C* with dental caries in permanent dentition of adults (N = 573, 25–50 years) and primary dentition of children (N = 449, 6–12 years).

		Permanent				Primary			
Gene	SNP	Phenotype	β estimate	SE	p-value	Phenotype	β estimate	SE	p-value
***KRT6A***	rs17845411 (K6a^N21S^)	DS	1.527	2.454	**0.0144**	ds	-0.126	-0.563	0.5734
		DMFS	3.502	2.505	**0.0125**	dfs	-0.404	-0.690	0.4904
		PF-DMFS	0.818	1.983	**0.0478**	pf-dfs	0.013	0.049	0.9606
	rs62617089 (K6a^R443W^)	DS	-2.968	-1.423	0.1552	ds	0.3068	0.451	0.6522
		DMFS	-7.3	-1.557	0.1199	dfs	-0.2014	-0.1131	0.9100
		PF-DMFS	-1.337	-0.9675	0.3337	pf-dfs	-0.5381	-0.6993	0.4847
	rs62617088 (K6a^V532I/F^)	DS	-3.962	-0.7643	0.4450	ds	-1.15	-0.6674	0.5048
		DMFS	24.88	2.142	**0.0326**	dfs	4.396	0.9748	0.3301
		PF-DMFS	4.09	1.193	0.2334	pf-dfs	4.702	2.030	**0.0430**
***KRT6C***	rs373213028 (K6c^Y62M/I^)	DS	-0.3984	-0.7322	0.4643	ds	-0.2299	-1.178	0.2392
		DMFS	-0.7495	-0.6127	0.5403	dfs	-0.09136	-0.1786	0.8583
		PF-DMFS	-0.04437	-0.1233	0.9019	pf-dfs	-0.1664	-0.7417	0.4587
	rs411107 (K6c^G88R^)	DS	-0.3576	-0.6481	0.5172	ds	-0.1539	-0.7534	0.4516
		DMFS	-0.6542	-0.5273	0.5982	dfs	0.3151	0.589	0.5561
		PF-DMFS	-0.07503	-0.2056	0.8372	pf-dfs	0.02438	0.1035	0.9176
	rs200653200 (K6c^G97R^)	DS	2.174	2.361	**0.0186**	ds	0.093	0.2671	0.7895
		DMFS	2.029	0.976	0.3294	dfs	-0.564	-0.618	0.5370
		PF-DMFS	0.844	1.380	0.1680	pf-dfs	-0.119	-0.299	0.7651
	rs394598 (K6c^G111D^)	DS	-0.07541	-0.1363	0.8916	ds	-0.1852	-0.9114	0.3625
		DMFS	-0.3352	-0.2695	0.7876	dfs	-0.2154	-0.4046	0.6859
		PF-DMFS	0.07602	0.2077	0.8355	pf-dfs	-0.2583	-1.11	0.2678
	rs151117600 (K6c^S143N^)	DS	1.552	2.479	**0.0185**	ds	-0.091	-0.403	0.6874
		DMFS	3.756	2.670	**0.0078**	dfs	-0.449	-0.763	0.4461
		PF-DMFS	0.855	2.060	**0.0399**	pf-dfs	-0.028	-0.109	0.9133
	rs11608915 (K6c^R182Q^)	DS	-0.7027	-1.313	0.1897	ds	0.05696	0.31	0.7567
		DMFS	-1.844	-1.534	0.1257	dfs	-0.05705	-0.1186	0.9057
		PF-DMFS	-0.1604	-0.4524	0.6512	pf-dfs	-0.2227	-1.068	0.2862
	rs412533 (K6c^V481I^)	DS	-0.4038	-0.7407	0.4592	ds	-0.2487	-1.281	0.2006
		DMFS	-8959	-0.7309	0.4651	dfs	-0.08172	-0.1606	0.8725
		PF-DMFS	-0.09512	-0.2637	0.7921	pf-dfs	-0.1556	-0.697	0.4862
	Rs71453291 (K6c^G538A^)	DS	-2.262	-0.9653	0.3348	ds	-1.169	-1.397	0.163
		DMFS	-6.548	-1.243	0.2143	dfs	-3.483	-1.591	0.1123
		PF-DMFS	-0.5726	-0.3688	0.7124	pf-dfs	-1.396	-1.514	0.1308
***KRT6B***	rs428894 (K6b^N21S^)	DS	-0.7057	-1.32	0.1874	ds	0.2388	1.291	0.1972
		DMFS	-1.626	-1.353	0.1766	dfs	0.2836	0.5851	0.5588
		PF-DMFS	-0.5035	-1.426	0.1543	pf-dfs	0.1425	0.671	0.5026
	rs144860693 (K6b^G97R^)	DS	1.626	2.611	**0.0093**	ds	-0.102	-0.452	0.6512
		DMFS	3.434	2.452	**0.0145**	dfs	-0.471	-0.800	0.4242
		PF-DMFS	0.809	1.958	**0.0507**	pf-dfs	-0.041	-0.161	0.8720
	rs61745883 (K6b^G111D^)	DS	0.7205	1.037	0.3000	ds	-0.3064	-1.123	0.2619
		DMFS	-0.946	-0.6055	0.5451	dfs	0.2102	0.294	0.7689
		PF-DMFS	-0.084	-0.1826	0.8552	pf-dfs	0.07402	0.2365	0.8182
	rs28538343 (K6b^S143N^)	DS	2.404	3.574	**0.0004**	ds	-0.106	-0.451	0.6525
		DMFS	4.276	2.816	**0.0050**	dfs	-0.387	-0.631	0.5286
		PF-DMFS	0.960	2.141	**0.0327**	pf-dfs	-0.101	-0.378	0.7058
	rs652423 (K6b^N227S/I^)	DS	-0.05969	-0.1027	0.9182	ds	-0.2959	-1.464	0.1438
		DMFS	-0.1918	-0.1469	0.8833	dfs	-0.0427	-0.08051	0.9359
		PF-DMFS	0.01969	0.05124	0.9591	pf-dfs	-0.1598	-0.6853	0.4935
	rs61746354 (K6b^Y497C^)	DS	-0.265	-0.213	0.8315	ds	1.047	2.182	**0.0296**
		DMFS	-1.116	-0.398	0.6905	dfs	2.917	2.322	**0.0206**
		PF-DMFS	-1.097	-1.343	0.1797	pf-dfs	1.742	3.152	**0.0017**
	rs61746355 (K6b^G499S^)	DS	-3.274	-1.395	0.1636	ds	0.2434	0.2896	0.7723
		DMFS	0.2415	0.04569	0.9636	dfs	-0.03597	-0.01634	0.987
		PF-DMFS	1.947	1.253	0.2109	pf-dfs	-0.1073	-0.1114	0.9114

Note: All models are adjusted for age and sex.

DS and ds, Number of tooth surfaces with untreated decay; DMFS and dfs, number of decayed, missing

due to decay, and filled surfaces; PF-DMFS and pf-dfs, partial DMFS and dfs limited to molars and premolars pit and fissure surfaces.

The SNPs identified in *KRT6A* and *KRT6C* were associated with increased caries experience in adults only. Among the missense polymorphisms identified in *KRT6B*, rs61746354 (K6b^Y497C^) was associated with higher caries experience in children, while rs144860693 (K6b^G97R^) and rs28538343 (K6b^S143N^) were associated with higher caries experience in adults (**Table 1**). These results indicate that the effect of specific polymorphisms in keratin genes may differ across dentition (primary vs. permanent).

Only one common missense SNP in *KRT16*, rs111383277 (*KRT16*:*c*.*121C>T;* K16p.Arg41Cys), was at a frequency higher than 1%, while none were found in *KRT17*. rs111383277 did not show significant association with dental caries experience in the cohorts tested. Due to this limited number of common SNPs in *KRT16* and *KRT17*, we were not able to conclude on the potential implication of these two keratins in caries risk.

### Caries-associated missense SNPs in *KRT6* genes result in substitutions in the head and tail domains of the proteins

Keratins are structured into three major domains with a central “rod” domain, directly involved in the dimerization and further assembly of keratin filaments, flanked by a “head” domain and a “tail” domain on the N-terminal side and C-terminal side, respectively (**Fig 1D**). Interestingly, all the missense polymorphisms that showed significant association with higher caries experience in *KRT6A*, *KRT6B* and *KRT6C* result in amino acid substitutions in the head or tail domains, while all the mutations that have been associated so far with PC are located at the beginning or at the end of the rod domain (**Fig 1D**). The *KRT6B* polymorphism associated with higher caries experience in children (rs61746354, K6b^Y497C^) is the only SNP that results in an amino acid substitution in the tail domain (**Fig 1D**). The missense SNPs in *KRT6* genes were present at various frequencies in the cohorts studied (**S1 Table**). Moderate to high linkage disequilibrium (R^2^ between 0.69 and 0.89) was observed between rs17845411 (K6a^N21S^), rs151117600 (K6c^S143N^), rs144860693 (K6b^G97R^), and rs28538343 (K6b^S143N^) (**Fig 1E**).

**Fig 2:**
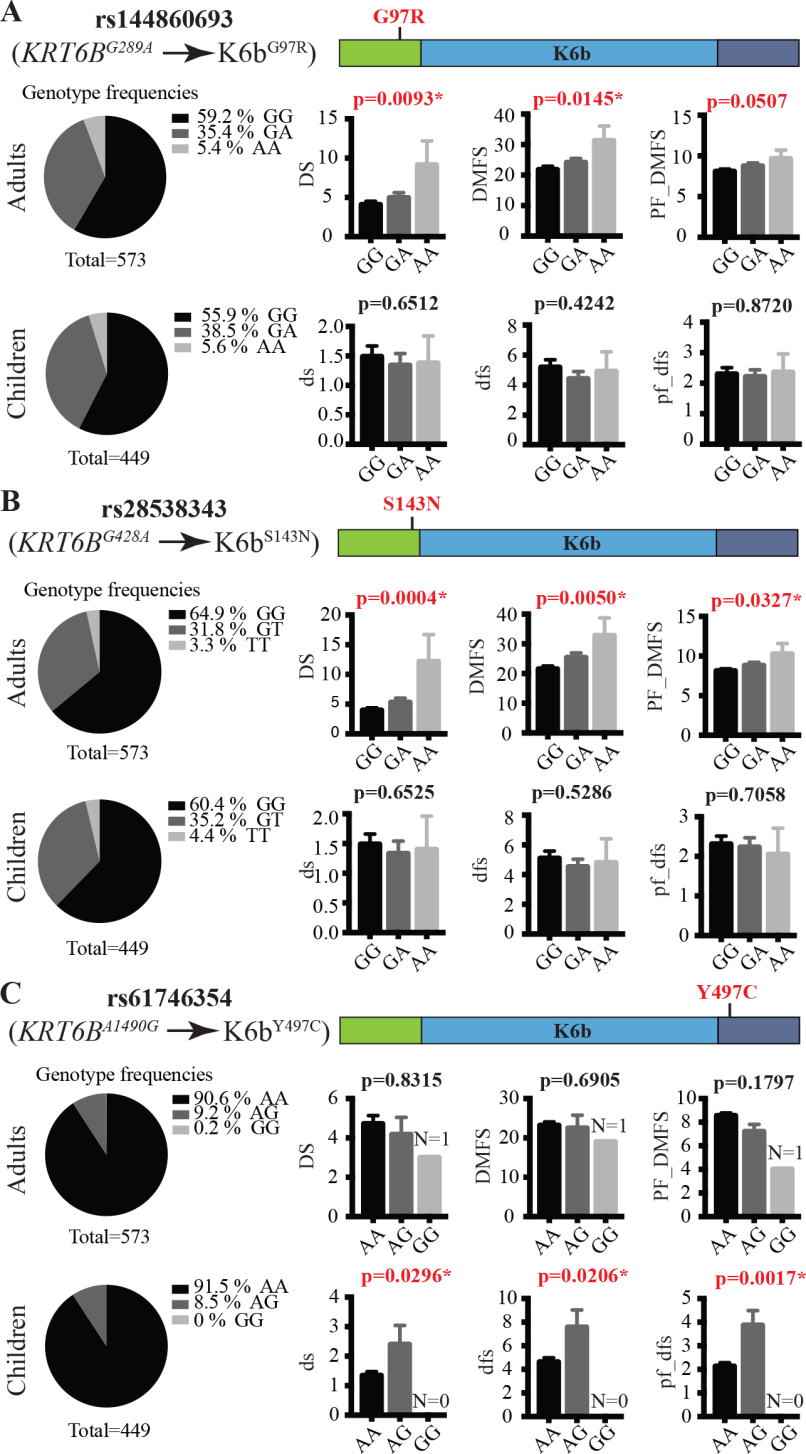
Genetic association between missense SNPs in *KRT6B* and tooth decay experience. Genotype frequencies and quantification of caries experience per genotype for missense SNPs rs144860693 (**A**), rs28538343 (**B**) and rs61746354 (**C**). Pie charts on the left show the frequencies of all three genotypes for each SNP in the cohorts of 573 adults and 449 children that were evaluated for their caries experience. Bar graphs on the right represent the average and SEM (error bar) measures of three indices standardly used to assess caries experience: left, the number of tooth surfaces with untreated decay (DS and ds); center, the number of decayed, missing due to decay, and filled surfaces (DMFS and dfs); right, partial DMFS and dfs indices limited to molars and premolars pit and fissure surfaces (PF_DMFS and pf_dfs).

Genotype frequencies and quantifications of caries experience per genotype group for the three missense SNPs identified in *KRT6B* are shown in **Fig 2**. The frequencies of rs144860693 (K6b^G97R^) and rs28538343 (K6b^S143N^), the two variants that exhibited the most significant association with dental caries risk in adults, are high in the cohorts studied (**Fig 2A and 2B; S1 Table**). These two SNPs have a major impact on caries experience, with an estimated increase in the average number of carious tooth surfaces of 1.6 and 2.4 surfaces per copy of the risk allele, respectively (**Table 1**). These variants did not demonstrate a statistically significant effect on average caries experience in children (**Fig 2A and 2B**). rs61746354 (K6b^Y497C^), the missense SNP in *KRT6B* that was associated with higher caries risk in children and occurs at a frequency higher that 4% in our cohorts (**S1 Table**), was associated with an estimated 1-surface increase in the average number of carious tooth surfaces (**Table 1 and Fig 2C**). Genotype frequencies and quantifications of caries experience per genotype for the other missense SNPs identified in *KRT6A* and KRT6*C* are shown in **S3 Fig**.

### Statistically significant interaction effects are observed between caries-associated missense SNPs in *KRT6* genes

Given that *KRT6B* harbors three missense SNPs showing significant association with caries experience, we wanted to further quantify the genetic relationship of missense variants in this gene on dental caries. To do so, we examined pairwise interactions between rs144860693 (K6b^G97R^), rs28538343 (K6b^S143N^) and rs61746354 (K6b^Y497C^). Statistically significant interaction effects were observed between rs144860693 (K6b^G97R^) and rs28538343 (K6b^S143N^) on the number of surfaces with untreated decay (DS) model, between rs144860693 (K6b^G97R^) and rs61746354 (K6b^Y497C^), and rs28538343 (K6b^S143N^) and rs61746354 (K6b^Y497C^) on the number of decayed, missing due to decay, and filled surfaces (DMFS), adjusting for age, sex, and all the other SNPs in *KRT6B* (**S2 Table**). Even though rs61746354 (K6b^Y497C^) was associated with higher caries risk in children only, this SNP exhibited a significant statistical interaction effect with rs144860693 (K6b^G97R^) and rs28538343 (K6b^S143N^) in adults (**S2 Table**). Therefore, the effect of the two SNPs that result in amino acid substitutions in the head domain of K6b on caries risk in adults may be influenced by the presence or absence of the p.Tyr497Cys substitution in the tail domain of the same keratin, a SNP that by itself is associated to higher caries risk only in children. The two SNPs resulting in p.Ser143Asn substitution in *KRT6B* and *KRT6C* (rs28538343 and rs151117600, respectively) also exhibited statistically significant interaction effect on the number of surfaces with untreated decay (DS) in adults (p-value = 0.044). When focusing on the 4 SNPs that lead to higher caries risk in adults, we found a significant cumulative effect of the number of risk alleles on caries experience (**S4 Fig**).

Given that *KRT75* is adjacent and phylogenetically related to the *KRT6* genes in the human genome, we explored potential linkage disequilibrium and interaction effects between the *KRT75* SNP previously shown to increase caries experience in adults [19] and the newly identified SNPs in *KRT6* genes. The previously reported SNP rs2232387 (K75^A161T^) was not in linkage disequilibrium with any of the *KRT6* SNPs (**S5 Fig**) and there was no statistical interaction (all p-values >0.05) between the same SNPs.

Altogether, our data support genetic association between SNPs in *KRT6A*, *KRT6B* and *KRT6C* and tooth decay risk, in a way that is dentition-specific, and with statistical interaction between various loci in these three genes.

### PC patient with mutation in *KRT6A* exhibits structural enamel defects

**Fig 3:**
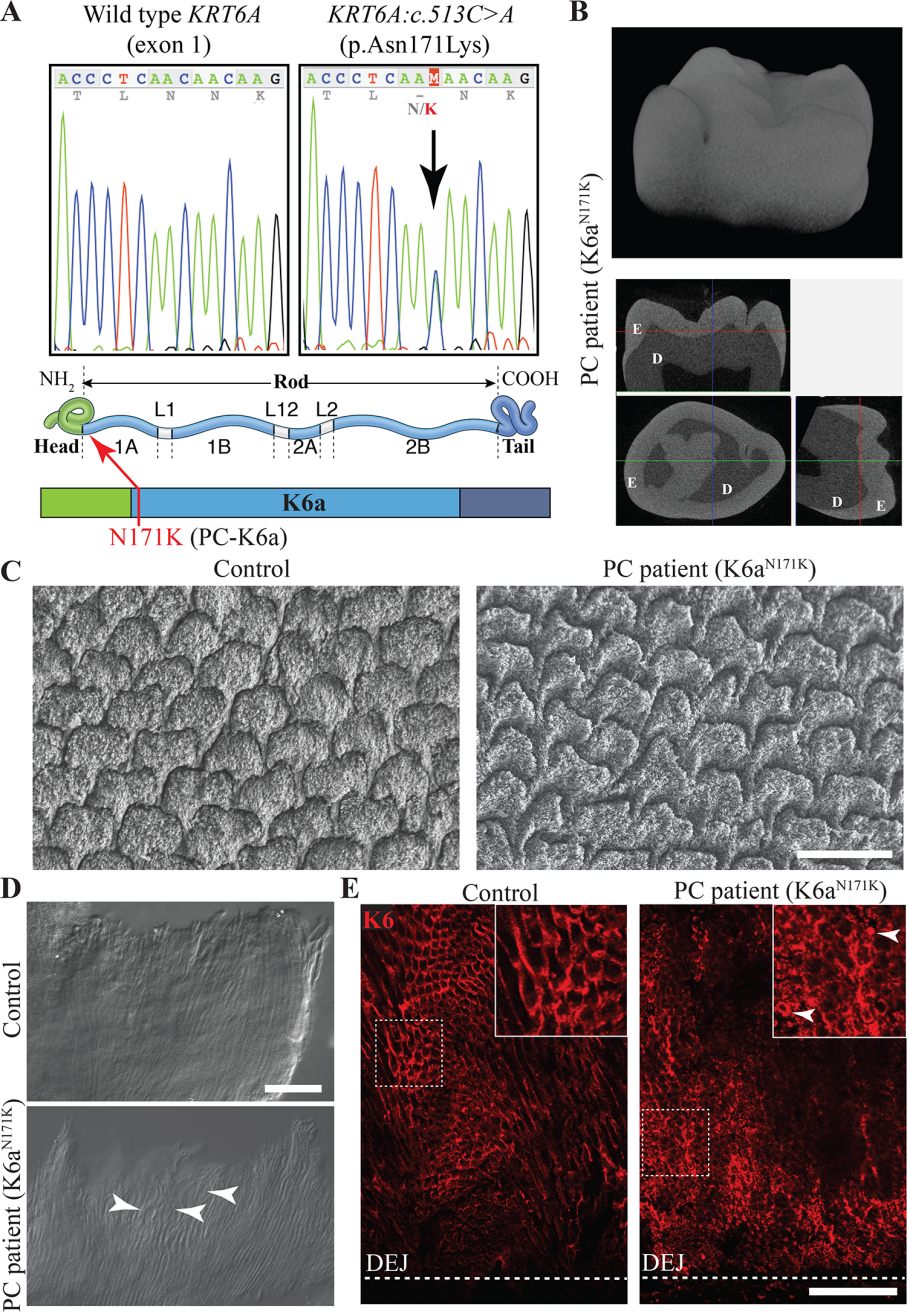
Structural enamel defects in third molars from a patient with PC-causing mutation in *KRT6A*. (**A**) Chromatograph on the left shows wild type sequence of *KRT6A* exon 1 at position c.505–519. Chromatograph on the right shows equivalent region for a PC patient with heterozygous mutation c.513C>A leading to missense mutation p.Asn171Lys (K6a^N171K^). Schematics in lower panel shows the position of the N171K amino acid substitution at the beginning of the rod domain in K6a. (**B**) Micro-computed tomography analysis of a wisdom tooth extracted from a PC patient who is heterozygous for the mutation described in A. The upper panel shows the 3D reconstruction of the tooth crown. The bottom panel shows 3-axes sections of the molar on which enamel (E) and dentin (D) can be distinguished. (**C**) Scanning electron microscopy analysis of polished transverse section of wisdom teeth from PC and control patients. Sections were taken in the area of the cusps and in a plane perpendicular to the orientation of the enamel rods. Scale bar: 10 μm. (**D**) Differential interference contrast imaging of insoluble organic material isolated from a third molar from PC and control patients. White arrowheads indicate curls formed by the rod sheaths. Scale bar: 50 μm. (**E**) Immunohistochemical detection of K6 (red) on polished section of human third molar extracted from PC and control patients. White arrowheads indicate aggregates. Scale bar: 50 μm. DEJ, dentin-enamel junction.

In order to assess how mutations in *KRT6* genes may affect enamel structure, we analyzed third molars that were extracted from a PC patient who is heterozygous for the *KRT6A*:*c*.*513C>A* transversion that results in p.Asn171Lys amino acid substitution (K6a^N171K^) (**Fig 3A**). This patient is a white male who was 18 years old at the time his third molars were extracted, and is the member of a family in which the mutation in *KRT6A* was previously reported [22]. The patient experienced 20/20 nail dystrophy, very painful palmoplantar keratoderma, oral leukokeratosis, follicular keratosis, but did not have natal teeth. The overall shape and structure of the third molar enamel did not appear defective based on micro-computed tomography analysis (**Fig 3B**). However, scanning electron microscopy analysis of polished sections of the teeth (section plane transverse to the enamel rods) revealed alteration of the distribution and shape of enamel rods when compared to third molars extracted from healthy patients (**Fig 3C**).

The insoluble organic material present in mature enamel can be isolated after full demineralization of a tooth in EDTA. When isolated from molars extracted from this PC patient, the insoluble material exhibited uneven alignment of the enamel rod sheaths that tended to form curls (**Fig 3D**). To assess the effects of the K6a^N171K^ mutant protein on K6 distribution in enamel, we performed immunohistochemical staining using anti-K6 antibody on polished sections of the patient’s tooth. K6 staining was still found to be stronger in the tuft areas near the DEJ (**Fig 3E**). However, K6 distribution was no longer restricted to the interrod but could also be found as smaller rings or clumps within rods (**Fig 3E**). These results indicate that this PC-causing mutation in *KRT6A* leads to improper incorporation of the K6a protein into enamel rod sheaths, which results in altered shape and arrangement of enamel rods.

### Caries-associated p.Ser143Asn and p.Tyr497Cys substitutions alter K6 protein behavior in ameloblast-like cells

**Fig 4:**
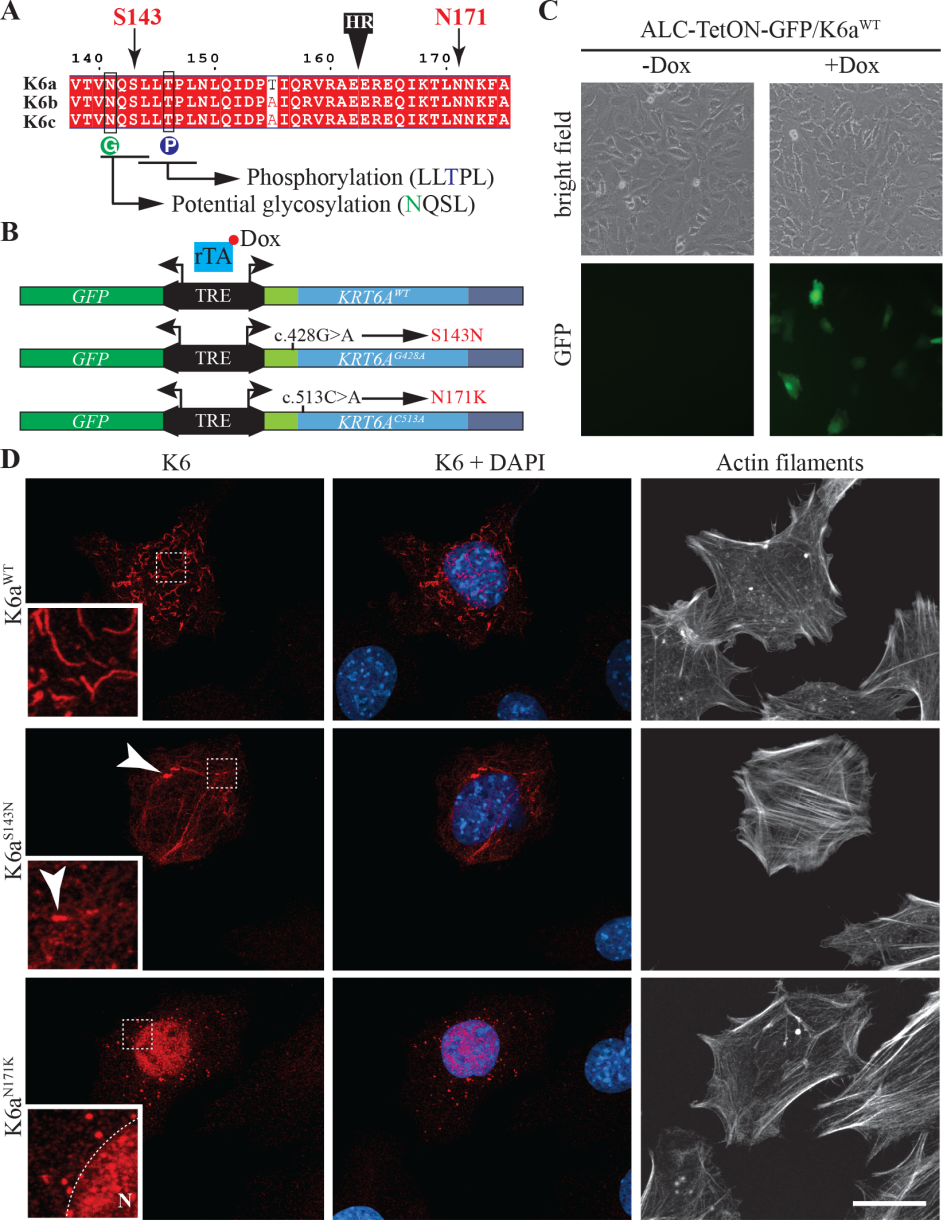
Effects of S143N and N171K mutations on the assembly of K6a in ameloblast-like cells. (**A**) Alignment of K6a, K6b and K6c protein sequences between amino acids 138 and 175 showing the position of serine 143 and asparagine 171 on both sides of the transition between the head and the rod domain (black box labeled HR). Serine 143 is right upstream of an LL^**S**^**/**_**T**_PL consensus phosphorylation site (blue circle labeled **P**) that is highly conserved in type II keratins. Serine 143 is also part of an **N**QSL potential N-glycosylation site (green circle labeled **G**). (**B**) Schematic representation of pBi4-GFP/K6a bidirectional constructs used for tetracycline inducible expression of GFP with *KRT6A*^*W*T^, *KRT6A*^*G428A*^ (produces K6a^S143N^) or *KRT6A*^*C513A*^ (produces K6a^N171K^ mutant). When transfected into cells expressing the transactivator rTA, transgene expression through binding of rTA to the tetracycline responsive element (TRE) can be induced by addition of doxycycline (Dox) to the culture medium. (**C**) Detection of GFP induction in ameloblasts-like cells stably expressing rTA (ALC-TetON) transfected with pBi4-GFP/K6a constructs and grown with or without Dox. (**D**) Immunohistochemical analysis of K6a subcellular distribution (red) in ALC-TetON cells producing K6a^WT^, K6a^S143N^ or K6a^N171K^. Nuclei are stained with DAPI (blue) and actin filaments are stained with phalloidin conjugated to Alexa Fluor 647 (white).

Missense mutations in keratins may affect their assembly, modify their subcellular localization and/or affect their interaction with keratin-associated proteins. Phosphorylation and glycosylation of the head and tail domain of intermediate filaments proteins have been shown to influence their interaction with other proteins and their subcellular localization [23, 24]. When comparing the position of the SNPs we determined to be associated with increased caries experience and potential sites for post-translational modifications in K6 proteins, we observed that the p.Ser143Asn substitution (rs28538343 in *KRT6B* and rs151117600 in *KRT6C*) is immediately adjacent to an LL^**S**^/_**T**_PL consensus phosphorylation site that is highly conserved in type II keratins [24, 25], and within a potential N-linked glycosylation site (**Fig 4A and S6 Fig**). Although it remains to be determined how K6 proteins interact with and are deposited into the enamel matrix in the context of a secretory stage ameloblast *in vivo*, we assessed the effect of the p.Ser143Asn substitution in K6 in a context in which the mutant protein is overexpressed in ameloblast-like cells (ALC) [26]. In this assay, we also analyzed the behavior of K6^N171K^ mutant protein carried by the PC patient included in this study (**Fig 3**), a mutation located in the rod domain and known to have a severe effect on keratin filament assembly [27, 28]. Given the high degree of sequence identity between K6 proteins (**S7 Fig**) and the fact that the mutations of interest are located in highly conserved regions (**Fig 4A**), we used K6a as a model protein for this assay. We used site-directed mutagenesis to introduce the *c*.*428G>A* transition (results in p.Ser143Asn substitution) and the *c*.*513C>A* transversion (results in p.Asn171Lys substitution) into the *KRT6A* cDNA, and cloned the different isoforms (*KRT6A*^*WT*^, *KRT6A*^*c*.*428G>A*^ and *KRT6A*^*c*.*513C>A*^) into a vector that allows for tetracycline inducible co-expression of *KRT6A* isoforms and *GFP* (**Fig 4B**).

These constructs were used to transfect ALC-TetON cells in which expression of *KRT6A* isoforms and *GFP* can be induced by addition of doxycycline to the culture medium (**Fig 4C**). Immunohistochemical analysis using anti-K6 antibody was used to determine the distribution of K6a isoforms in ALC-TetON cells (**Fig 4D**). While K6a^WT^ formed thick and relatively short bundles of keratin filaments in ALC-TetON cells, K6a^S143N^ tended to form a web of thinner filaments together with large aggregates (**Fig 4D**). These large aggregates were not seen with K6a proteins harboring the p.Asn21Ser and p.Gly97Arg substitutions (S8A and S8B Fig) caused by the other SNPs that are associated with higher caries in adults (rs17845411 and rs144860693, respectively) and are in partial linkage disequilibrium with the SNPs leading to p.Ser143Asn substitution (rs28538343 in *KRT6B* and rs151117600 in *KRT6C*). These results suggest that the p.Ser143Asn substitution may contribute most significantly to the caries-prone phenotype in adults. The behavior of the K6a^N171K^ mutant protein fused to a YFP tag has been previously studied in the context of human hepatoma PLC cells in which the mutant protein was shown to form aggregates primarily located in the cytoplasm [27, 28]. In the context of ALC-TetON cells, K6a^N171K^ formed aggregates that showed heightened accumulation in the nucleus (**Fig 4D**). These results confirm a severe impairment of K6a assembly in PC patients with p.Asn171Lys substitution. The fact that the aggregates in PLC cells were mostly in the cytoplasm may reflect a cell-specific behavior of the mutant protein or may be due to the YFP tag that was fused to K6a in these experiments [27, 28].

Given that the SNP leading to p.Tyr497Cys substitution in the tail domain of K6b (rs61746354) is the only one we found associated with higher caries risk in children, we tested its effect on K6a assembly. Similarly to K6a^S143N^, the K6a^Y497C^ isoform tended to form large aggregates in ALC-TetON cells (S8C Fig), which suggests that this SNP may be the cause of the caries-prone phenotype in children. Even though this substitution is not found near a potential posttranslational modification site, the presence of a new cysteine in the tail domain may result in the formation of disruptive disulfide bonds. Given the interaction effects measured between the SNPs that lead to the p.Ser143Asn and p.Tyr497Cys substitutions, we generated a DNA construct for the expression of a K6a protein that harbors both substitutions (K6a^S143N-Y497C^). This double mutant tends to form aggregates to the same extent as the single mutant proteins (S8D Fig).

Taken together, these results confirm that the PC-associated p.Asn171Lys substitution results in profound impairment of K6a protein assembly, and reveal that the caries-associated p.Ser143Asn and p.Tyr497Cys substitutions in K6 proteins also affect the behavior of the proteins, when overexpressed in an ameloblast cell line.

## Discussion

The present report highlights the contribution of specific sets of keratins to the organic fraction of mature tooth enamel and demonstrates through genetic and analytical studies their crucial function in the formation of enamel and its resistance to decay. K75, an epithelial hair keratin in which mutations have been associated with hair disorders, was the first keratin we investigated in this context [19]. K6 proteins, that are the focus of the present study, are expressed in epithelia that withstand particularly high levels of mechanical strain (palmoplantar skin, oral epithelium) as well as in the supporting layers of the hair follicle where their function is similar to that of K75 which is not expressed in palmoplantar epidermis and oral epithelium. Our findings demonstrate that, as K75, K6 proteins play a crucial role in the enamel rod sheath and that mutations in the genes encoding these keratins may impair the stability of the organic structural component of mature enamel. We propose that, with their unique biochemical properties, K75 and K6 contribute to the toughness, elasticity and resistance to degradation of the enamel rod sheaths, which contributes to establishing proper shape and arrangement of enamel rods and enhances the biomechanical properties of tooth enamel [12, 13]. Moreover, since it has long been suggested that the stability of the proteins in mature enamel influences the resistance of enamel to carious attack [14, 15], we propose that keratins contribute to the stability of enamel rod sheaths and therefore to the resistance of enamel to decay.

Since K6 proteins are also expressed in the oral epithelium, and patients with PC may exhibit oral leukokeratosis, there could be a partial involvement of the oral cavity in the increased susceptibility to caries measured in this study. However, the fact that we found SNPs that lead to a higher number of caries in children and not in adults (same oral cavity but different set of teeth) strongly suggests that defects in the dental tissue itself are the major factor leading to this effect. The structure and chemical composition of tooth enamel is known to be different between primary and permanent teeth. Primary teeth exhibit thinner and whiter enamel with a smoother surface and higher content in calcium and phosphate when compared to permanent teeth [29, 30]. Enamel from primary teeth also has a greater susceptibility to demineralization [31]. Moreover, it has been proposed that the genetic factors influencing dental caries differ between primary and permanent dentition [9]. However, there has been no study comparing the composition of the organic material present in the enamel from primary and permanent teeth. The dentition-specific effect we report here for SNPs in KRT6 genes, which we previously observed for two SNPs in *KRT75*, with one affecting adults and the other one affecting children [19], suggests that the combination and/or the mode of incorporation of these keratins in the enamel rod sheaths is different in primary and permanent teeth.

Even though this is the first evidence of K6 proteins being incorporated into the enamel matrix, a previous yeast-two-hybrid study determined that K6 could interact with enamel matrix proteins such as amelogenin and tuftelin [32]. It is therefore likely that K6 proteins interact with enamel matrix proteins during the process of enamel secretion. However, the mode of incorporation of keratins into the enamel matrix remains to be elucidated. The interaction of keratins with other proteins is known to involve their head and tail domains rather than the rod domain through which heterodimerization of acidic and basic keratins is established. These interactions are regulated by posttranslational modifications such as phosphorylation and glycosylation [23, 24], and mutations impairing such modifications have been linked to skin diseases [33], as well as diseases related to liver and pancreatic injury [34]. Interestingly, all the caries-associated missense SNPs we identified in *KRT6* genes result in substitutions in the head and tail domains of the proteins, which suggests that they may affect their interaction with other proteins rather than their heterodimerization. We further demonstrate that the p.Ser143Asn substitution that may affect phosphorylation and/or glycosylation of the head domain of K6 proteins affects the behavior of K6A in the context of an ameloblast cell line, which suggests that this substitution found in both K6b and K6c may contribute most significantly to the caries-prone phenotype in adults. Functional studies will be required to elucidate the effects of the p.Ser143Asn substitution on the biochemical properties of K6 proteins, in particular on its ability to undergo posttranslational modifications that may affect interaction with enamel matrix proteins and incorporation into the enamel *in vivo*. Interestingly, in a recent clinical report, an isolated case of PC was proposed to be caused by de novo c.428G>A mutation in *KRT6A* that leads to the p.Ser143Asn substitution [35]. This is so far the only report of PC-causing mutation outside of the rod domain. Based on its location in the head domain and on the high frequency of the same substitution in K6b and K6c, the p.Ser143Asn substitution in K6a is unlikely to be the sole cause for the PC phenotype in this patient.

In epidermal tissues, K6 proteins (Type II, basic or neutral) form heterodimers with K16 or K17 (Type I, acidic) to assemble in larger polymeric structures. Interestingly, the subcellular distribution of K16 and K17 proteins is distinct from the distribution of K6 in the enamel organ, which suggests that they do not follow their canonical mode of assembly in this tissue. Due to the low number of frequent missense SNPs in *KRT16* and *KRT17* in our cohorts, the present study did not allow us to make any conclusion on the potential genetic association between variants in these two genes and dental caries experience. However, the striking difference in the way K16 and K17 proteins are incorporated into enamel suggests that their function in this tissue is distinct from that of K6 proteins. Based on the restricted localization of K16 and K17 near the DEJ and in the core of the enamel rods, these keratins may be involved in shock absorption and protection against fracture [13] rather than in the resistance to caries. Structural analysis of enamel from PC patients with mutations in *KRT16* and *KRT17* will help address this question.

In conclusion, we show for the first time that (i) K6 proteins are incorporated into mature tooth enamel at the rod sheaths, (ii) SNPs in *KRT6* genes are associated with increased susceptibility to dental caries, (iii) a PC patient with a mutation in *KRT6A* exhibits defects in enamel structure, and (iv) caries-associated p.Ser143Asn substitution in K6 proteins impairs proper protein interactions.

## Materials and methods

### Ethics statement

We thank the Pachyonychia Congenita Project and Ms. Holly Evans for providing us with clinical information and extracted third molars from a PC patient (20040468–1057496), and the NIDCR dental clinic for providing extracted third molars from healthy patients (NCT01805869).

For the COHRA study, written informed consent was provided by all adult participants, and verbal assent with parental written consent was provided by all child participants. All procedures, forms and protocols were approved by the Institutional Review Boards of the University of Pittsburgh and West Virginia University.

Written informed consent was obtained from the pachyonychia congenita patient, as part of a research registry approved by an institutional review board that complies with all principles of the Helsinki Accord (Western IRB study no. 20040468).

All animal work was approved by the NIAMS Animal Care and Use Committee.

### Human subject recruitment for the COHRA study

The Center for Oral Health Research in Appalachia (COHRA) study was initiated to investigate the community-, family-, and individual-level contributors to oral health [36]. Participants from rural counties of Pennsylvania and West Virginia were enrolled via a household-based recruitment strategy, with eligible households required to include at least one biological parent-child pair. All other members of eligible households were invited to participate without regard to biological or legal relationships, or oral health status. Written informed consent was provided by all adult participants, and verbal assent with parental written consent was provided by all child participants. All procedures, forms and protocols were approved by the Institutional Review Boards of the University of Pittsburgh and West Virginia University.

### Data collection for the COHRA study

Intra-oral examinations of all participants were performed by licensed dentists and/or research dental hygienists. Each surface of each tooth (excluding third molars) was examined for evidence of decay, from which dental caries indices were generated. Three measurements of caries experience were considered: (1) the number of surfaces with untreated decay (DS/ds); (2) the traditional DMFS/dfs indices which represent the number of decayed (D/d), missing due to decay (M), and filled (F/f) tooth surfaces (S/s) in the permanent (DMFS) and primary (dfs) dentitions; and (3) the partial DMFS and dfs indices limited to the molars and premolar pit and fissure surfaces which are at high risk of decay. DNA samples were collected via blood, saliva or buccal swab. Genotyping for approximately 600,000 polymorphisms was performed by the Center for Inherited Disease Research at Johns Hopkins University using the Illumina Human610-Quadv1_B BeadChip (Illumina). Extensive data cleaning and quality assurance analyses were performed as previously reported [4]. Imputation of approximately 16 million unobserved genetic variants was performed using the 1000 Genome Project (phase 1 June 2011 release) as reference. In brief, pre-phasing of haplotypes was performed via SHAPEIT2 [37] and imputation was performed via IMPUTE2 [38].

### Statistical analysis for the COHRA study

Linear regression was used to test the association of dental caries experience with genetic polymorphisms under the additive genetic model while adjusting for age and sex. Pairwise SNP-by-SNP interaction effects were tested in the same modeling framework by including main effects of each SNP and their product term for selected variants within the same gene region, along with age and sex. Analyses were performed separately for dental caries in the permanent dentition of adults (ages 25–50 years) and the primary dentition of children (ages 6–12 years). All analyses were limited to self-reported non-Hispanic whites (mixed European descent); self-reported race was consistent with genetically-determined ancestry. Analyses were performed using PLINK (v1.9) (http://www.cog-genomics.org/plink/1.9/)[39] and R (R Foundation for Statistical Computing).

### Collection of third molars from patient with pachyonychia congenita

Third molars were obtained from a patient involved in the International Pachyonychia Congenita Research Registry (IPCRR), under the IRB Protocol number 20040468 and IRB Study number 1057496. Third molars from healthy patients were obtained from the NIDCR OP-1 Dental Clinic that were collected under the IRB Protocol number NCT01805869.

### RNA-seq analysis

RNA-seq analysis was performed as described previously [40]. Briefly, mandibles were dissected from P10 mice, transferred to RNAlater solution (Life Technologies) and stored at 4°C. Enamel organs were dissected from mandibles and homogenized in Trizol reagent (Invitrogen) using the Precellys 24 (Bertin Technologies). Total RNA was extracted and further purified using the RNAeasy mini kit (Qiagen). RNA-seq analysis was performed using the Mondrian SP kit (Illumina) and the Illumina HiSeq 2000 sequencing system.

### Immunohistochemical analysis on rat mandible section

Rat mandibles at postnatal day 10 were fixed overnight at 4°C in 4% paraformaldehyde in 1X PBS, dehydrated and embedded in paraffin and 10 μm-thick sections were prepared. Immunohistochemical analysis was performed using a blocking solution containing 5% goat serum and 7.5% BlockHen II (Aves Labs, Tigard, OR) in 1X PBS. Enzymatic antigen retrieval was performed using Ultravision Trypsin (Thermo Fisher Scientific, Waltham MA). Primary antibodies used: Guinea-pig anti-K6 (Progen Biotechnik GmbH, Germany), guinea-pig anti-K17 (Progen Biotechnik GmbH, Germany). Alexa-488 anti-guinea-pig (Thermo Fisher Scientific, Waltham MA) was used as secondary antibody. Images were acquired on a Leica LS5 confocal microscope (Leica Microsystems Inc., Buffalo Grove, IL).

### Immunohistochemical analysis on polished human tooth sections

Ground, polished and etched human teeth were stained with guinea-pig anti-K6 (Progen Biotechnik GmbH, Germany), mouse anti-K6 (Abcam, Cambridge MA), guinea-pig anti-K16 (Progen Biotechnik GmbH, Germany) or guinea-pig anti-K17 (Progen Biotechnik GmbH, Germany) antibody. Alexa-488 anti-guinea-pig and Alexa-555 anti-mouse (Thermo Fisher Scientific, Waltham MA) were used as secondary antibodies.

### Scanning electron microscopy

Ground, polished and etched human teeth were prepared for SEM as described previously [19]. Samples were fixed overnight at 4°C in 2% glutaraldehyde, 2% PFA in 0.1M phosphate buffer pH 7.4 and dehydrated through a series of 50%, 70%, 95% and 100% ethanol solutions. They were incubated for 10 min in hexamethyldisilazane, air-dried for 30 min, mounted on aluminum specimen mount stubs covered with conductive carbon adhesive tabs (Electron Microscopy Sciences, Hatfield, PA), sputter-coated with gold and analyzed under a Field Emission Scanning Electron Microscope S4800 (Hitashi, Toronto, Canada) at 10 kV.

### Micro-CT analysis and 3-D reconstructions

Micro-CT analysis of fixed molars was performed as described previously [19] using the Skyscan 1172 desktop X-ray microfocus CT scanner and the following parameters: 0.5mm AI + 0.1mm Cu filters, 100 kV, 100 uA, 8.00 micron resolution, 0.4 degrees rotation step over 180 degrees). CTvox software (Bruker microCT) was used for 3D reconstruction.

### Plasmids

The cDNAs encoding the, K6a^N21S^, K6a^G97R^, K6a^S143N^, K6a^Y497C^ and K6a^N171K^ mutant proteins were generated by site-directed mutagenesis using the QuikChange Site-directed Mutagenesis Kit (Stratagene). The following primers were used: N21S-forward: GGGGTTTCAGTGCCAgCTCAGCCAGGC, N21S-reverse: GCCTGGCTGAGcTGGCACTGAAACCCC, G97R-forward: GGCTTTGGTGGCGCCaGGAGTGGATTGG, G97R-reverse: CCAATCCACTCCtGGCGCCACCAAAGCC, S143N-forward: GTCAACCAGAaTCTCCTGACTCCCCTC, S143N-reverse: GAGGGGAGTCAGGAGAtTCTGGTTGAC, Y497C-forward: CCGTCTCCAGTGGCTgTGGCGGTGCCAG, Y497C-reverse: CTGGCACCGCCAcAGCCACTGGAGACGG, N171K-forward: GATCAAGACCCTCAAaAACAAGTTTGCC, N171K-reverse: GGCAAACTTGTTtTTGAGGGTCTTGATC (lower cases indicate the position of the mutations). The pBi4-GFP vector was used to simultaneously express the reporter protein EGFP with K6a^WT^, K6a^N21S^, K6a^G97R^, K6a^S143N^, K6a^Y497C^, K6a^S143N-Y497C^, or K6a^N171K^ under control of a unique tetracycline responsive element.

### Cell culture and transfections

Murine Ameloblast-like cells (ALC) were kindly provided by Dr. Sugiyama [26] and used to produce a tetracycline inducible ameloblast cell line. These cells were stably transfected with a prtTA2-M2/IRES-Neo plamid obtained after subcloning of the rtTA2-M2 cassette [41] into the pCMV-IRES-Neo (Clontech). rtTA-M2 is a mutagenized form of rtTA that shows a lower basal activity and a higher sensitivity to doxycycline (Dox) than the original rtTA [41]. The presence of the IRES cassette before the neomycin (Neo) resistance gene allowed coexpression of the rtTA-M2 transactivator and the Neo resistance gene, increasing the chance to select clones that express sufficient amounts of the transactivator in Neo-resistant cells. Clones were isolated and functionality of the tet system was screened by transient transfection with pTRE2-luc (expression of luciferase under the control of tetracycline responsive element). Cells were grown in the presence or absence of 2 ug/ml Dox and luciferase activity was estimated. One clone exhibiting a low basal activity of the transactivator in the absence of Dox and a strong induction in the presence of Dox (+Dox/-Dox ratio) was selected for subsequent experiments and named ALC-TetON. ALC-TetON cells were grown in Dulbecco’s modified Eagle’s medium (10% fetal bovine serum, 1% penicillin/streptomycin and 1 ug/ml G418). For transfections, the cells were grown to at least 70% confluence. 2 million cells were used per transfection with either the pBi4-GFP/K6a^WT^, pBi4-GFP/K6a^N21S^, pBi4-GFP/K6a^G97R^, pBi4-GFP/K6a^S143N^, pBi4-GFP/K6a^Y497C^, pBi4-GFP/K6a^S143N-Y497C^, or pBi4-GFP/K6a^N171K^ plasmid and the pCMV-K16^WT^ plasmid (Amaxa Nucleofactor).

### Immunocytochemistry

Transfected cells were seeded on glass coverslips coated with 0.1% gelatin and immediately induced with 2ug/ml doxycycline. Twenty-four hours after induction, cells were washed three times in 1X PBS and fixed with 4% paraformaldehyde in PBS for 15 min at room temperature. A 5 min incubation in 0.2% Triton in 1X PBS was used to permeabilize the cells before blocking unspecific sites using 3% BSA in PBS for 1 h. Primary antibodies diluted in blocking solution were applied for 1 h. Primary antibody used: guinea-pig anti-K6 (Progen). Secondary antibodies diluted in blocking solution were applied for 30 min. Secondary antibodies used: Alexa Fluor 555 anti-guinea pig IgG (Life Technologies). Nuclei were stained using DAPI and coverslips were mounted on glass slides using Mowiol (Calbiochem). Images were acquired using a LEICA Sp5 confocal microscope.

## Supporting information

S1 Table Allele frequencies of missense SNPs in *KRT6A*, *KRT6B* and *KRT6C* that show significant association with dental caries experience. (DOCX)

S2 Table Interaction effects in *KRT6B*. (DOCX)

S1 Fig Incorporation of K16 and K17 into mature enamel. (**A**) Immunohistochemical analysis of K17 distribution in rat enamel organ at postnatal day 10. Nuclei are stained with DAPI (blue). Inset at the top shows enlarged view of Tomes' processes. PL, papillary layer; SI, stratum intermedium; Am, ameloblasts; TP, Tomes’ processes; R, rod; IR, interrod. Scale bar: 50 μm. (**B**) Immunohistochemical detection of K16 and K17 on polished sections of human third molars. As indicated by the schematics on the left, the images were acquired from mesial-distal sections of human third molars and show the area of enamel adjacent to dentin. DEJ, dentin-enamel junction. Scale bars: 100 μm.
(TIF)

S2 Fig Detection of K6 into mature enamel validated by two independent antibodies. Immunohistochemical detection of K6 on polished sections of human third molars. Two different antibodies against K6 (recognize K6a, K6b and K6c in humans) were used: a polyclonal antibody raised in guinea-pig against the C-terminus of the protein (green) and a monoclonal antibody raised in mouse against the N-terminus of the protein (red). As indicated by the schematics on the left, the images in the upper panels were acquired from mesial-distal sections of human third molars and show the area of enamel adjacent to dentin, whereas the images in the lower panels were acquired from transverse section of the crown near the cusps. DEJ, dentin-enamel junction (dashed lines). Scale bars: 100 μm for upper panels, 20 μm for lower panels.
(TIF)

S3 Fig Genetic association between missense SNPs in *KRT6A* and *KRT6C* and tooth decay experience. Genotype frequencies and quantification of caries experience per genotype for missence SNPs rs17845411 and rs151117600 (locations shown on the left). Pie charts in the center show the frequencies of all three genotypes for each SNP in the cohorts of 573 adults and 449 children that were evaluated for their caries experience. Bar graphs on the right represent the average and SEM (error bar) measures of three incices standardly used to assess caries experience: left, the number of tooth surfaces with untreated decay (DS and ds); center, the number of decayed, missing due to decay, and filled surfaces (DMFS and dfs); right, partial DMFS and dfs indices limited to molars and premolars pit and fissure surfaces (PF_DMFS and pf_dfs).
(TIF)

S4 Fig Cumulative effect of risk alleles in *KRT6A*, *KRT6B* and *KRT6C* on caries number. Number of caries for three phenotypes measured in adults (DS, DMFS, PF_DMFS) were plotted against the number of risk alleles carried for 4 SNPs in KRT6A, KRT6B and KRT6C that are associated with higher caries risk: rs17845411 (K6aN21S; risk allele: G), rs144860693 (K6bG97R; risk allele: A), rs28538343 (K6bS143N; risk allele A), and rs151117600 (K6cS143N; risk allele: A).
(TIF)

S5 Fig Linkage disequilibrium between caries risk-associated SNP in *KRT75* and SNPs in *KRT6A*, *KRT6B* and *KRT6C*. Linkage disequilibrium data measured by R2 between rs2232387, a missense SNP in KRT75 that was previously associated with higher caries risk, and five SNPs associated with higher caries risk in KRT6A, KRT6B and KRT6C. The KRT75 gene is located right downstream of KRT6B on chromosome 12. rs2232387 is a c.481G>A transition in KRT75 that causes an alanine-to-threonine substitution at position 161 (K75A161T).
(TIF)

S6 Fig Prediction of N-glycosylation sites in K6a. The presence of potential N-Glycosylation sites in K6a was determined using NetNGlyc 1.0 Server. (**A**) A potential glycosylation site was found for the arginine at position 141 in a consensus NQSL site that includes serine 143. (**B**) The potential glycosylation site at position 141 was not called when serine 143 was replaced with an arginine.
(TIF)

S7 Fig Alignment of K6a, K6b and K6c protein sequences. (TIF)

S8 Fig Effect of caries-associated SNPs on K6a assembly in ALC-TetON cells. Immunohistochemical analysis of K6a subcellular distribution (red) in ALC-TetON cells producing K6aN21S (A), K6aG97R (B), K6aY497C (C) or K6aS143N-Y497C (D). Nuclei are stained with DAPI (blue), actin filaments are stained with phalloidin conjugated to Alexa Fluor 647 (grey), and transfected cells express GFP (green). Scale bar: 20 μm.
(TIF)
