# Correction: Genetic variants in pachyonychia congenita-associated keratins increase susceptibility to tooth decay

**DOI:** 10.1371/journal.pgen.1008230

**Published:** 2019-06-24

**Authors:** Olivier Duverger, Jenna C. Carlson, Chelsea M. Karacz, Mary E. Schwartz, Michael A. Cross, Mary L. Marazita, John R. Shaffer, Maria I. Morasso

There are errors in the labelling in Fig 2. The authors have provided a corrected version here.

**Fig 2:**
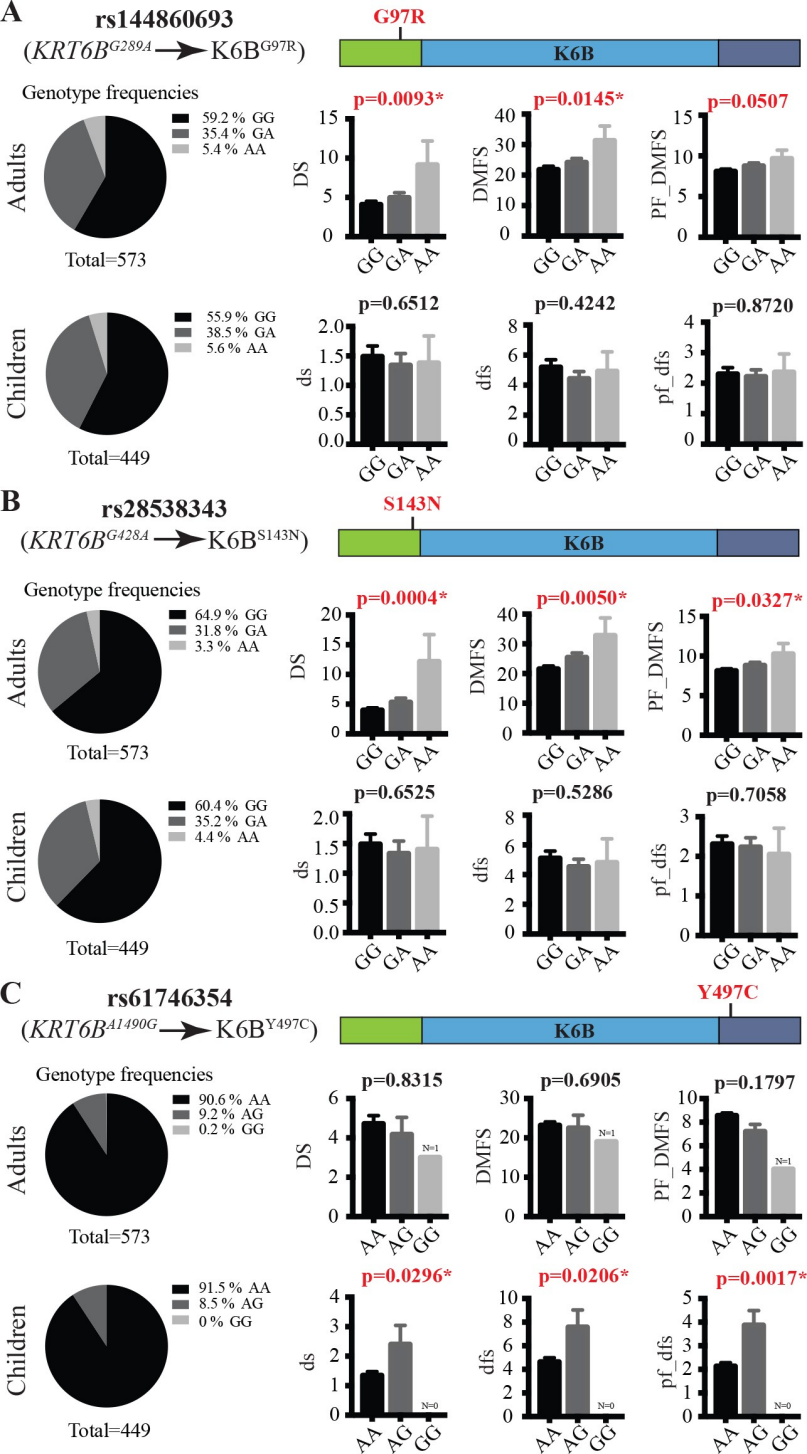
Genetic association between missense SNPs in *KRT6B* and tooth decay experience. Genotype frequencies and quantification of caries experience per genotype for missense SNPs rs144860693 (**A**), rs28538343 (**B**) and rs61746354 (**C**). Pie charts on the left show the frequencies of all three genotypes for each SNP in the cohorts of 573 adults and 449 children that were evaluated for their caries experience. Bar graphs on the right represent the average and SEM (error bar) measures of three indices standardly used to assess caries experience: left, the number of tooth surfaces with untreated decay (DS and ds); center, the number of decayed, missing due to decay, and filled surfaces (DMFS and dfs); right, partial DMFS and dfs indices limited to molars and premolars pit and fissure surfaces (PF_DMFS and pf_dfs).
