# Supplementary material for: Genetic variants in pachyonychia congenita-associated keratins increase susceptibility to tooth decay
Source: PLoS Genet. 2018 Jan 22;14(1):e1007168. doi: 10.1371/journal.pgen.1007168 (PMC5794186; doi:10.1371/journal.pgen.1007168)
Supplement: S1 Table — (DOCX) [file pgen.1007168.s001.docx]

**S1 Table:** Allele frequencies of missense SNPs in *KRT6A*, *KRT6B* and *KRT6C*

that show significant association with dental caries experience**.**

| SNP  (AA substitution) | Position  (transcript) | Major  allele | Minor  allele | Minor Allele Frequency | |
| --- | --- | --- | --- | --- | --- |
|  |  |  |  | Adults  (N=573) | Children  (N=449) |
| rs17845411 (K6a^N21S^) | 271 | A | G | 23.9% | 25.3% |
| rs151117600 (K6c^S143N^) | 476 | G | A | 23.1% | 24.8% |
| rs144860693 (K6b^G97R^) | 337 | G | A | 23.1% | 24.8% |
| rs28538343 (K6b^S143N^) | 476 | G | A | 19.2% | 22.0% |
| rs61746354 (K6b^Y497C^) | 1538 | A | G | 4.7% | 4.2% |
