# Supplementary material for: Genetic variants in pachyonychia congenita-associated keratins increase susceptibility to tooth decay
Source: PLoS Genet. 2018 Jan 22;14(1):e1007168. doi: 10.1371/journal.pgen.1007168 (PMC5794186; doi:10.1371/journal.pgen.1007168)
Supplement: S2 Table — (DOCX) [file pgen.1007168.s002.docx]

**S2 Table:** Interaction effects in *KRT6B*.

| Parameter | DS | | DMFS | |
| --- | --- | --- | --- | --- |
|  | β | p-value | β | p-value |
| Age | -0.2304 | <.0001 | 0.3396 | 0.0098 |
| Sexe | 3.0627 | <.0001 | -0.5921 | 0.7325 |
| rs144860693 (K6b^G97R^) | -2.0207 | 0.1395 | 1.8225 | 0.5510 |
| rs28538343 (K6b^S143N^) | 0.8177 | 0.6803 | 0.7404 | 0.8676 |
| rs61746354 (K6b^Y497C^) | -0.5720 | 0.7058 | 0.7565 | 0.8234 |
| rs144860693*rs28538343 (K6b^G97R^*K6b^S143N^) | 2.3164 | **0.0568** | 0.8979 | 0.7410 |
| rs144860693*rs61746354 (K6b^G97R^*K6b^Y497C^) | -1.3574 | 0.8363 | -56.2453 | **0.0001** |
| rs28538343*rs61746354 (K6b^S143N^*K6b^Y497C^) | 3.2624 | 0.6216 | 52.7658 | **0.0004** |
