# Supplementary figures and images for: Genetic variants in pachyonychia congenita-associated keratins increase susceptibility to tooth decay
Source: PLoS Genet. 2018 Jan 22;14(1):e1007168. doi: 10.1371/journal.pgen.1007168 (PMC5794186; doi:10.1371/journal.pgen.1007168)

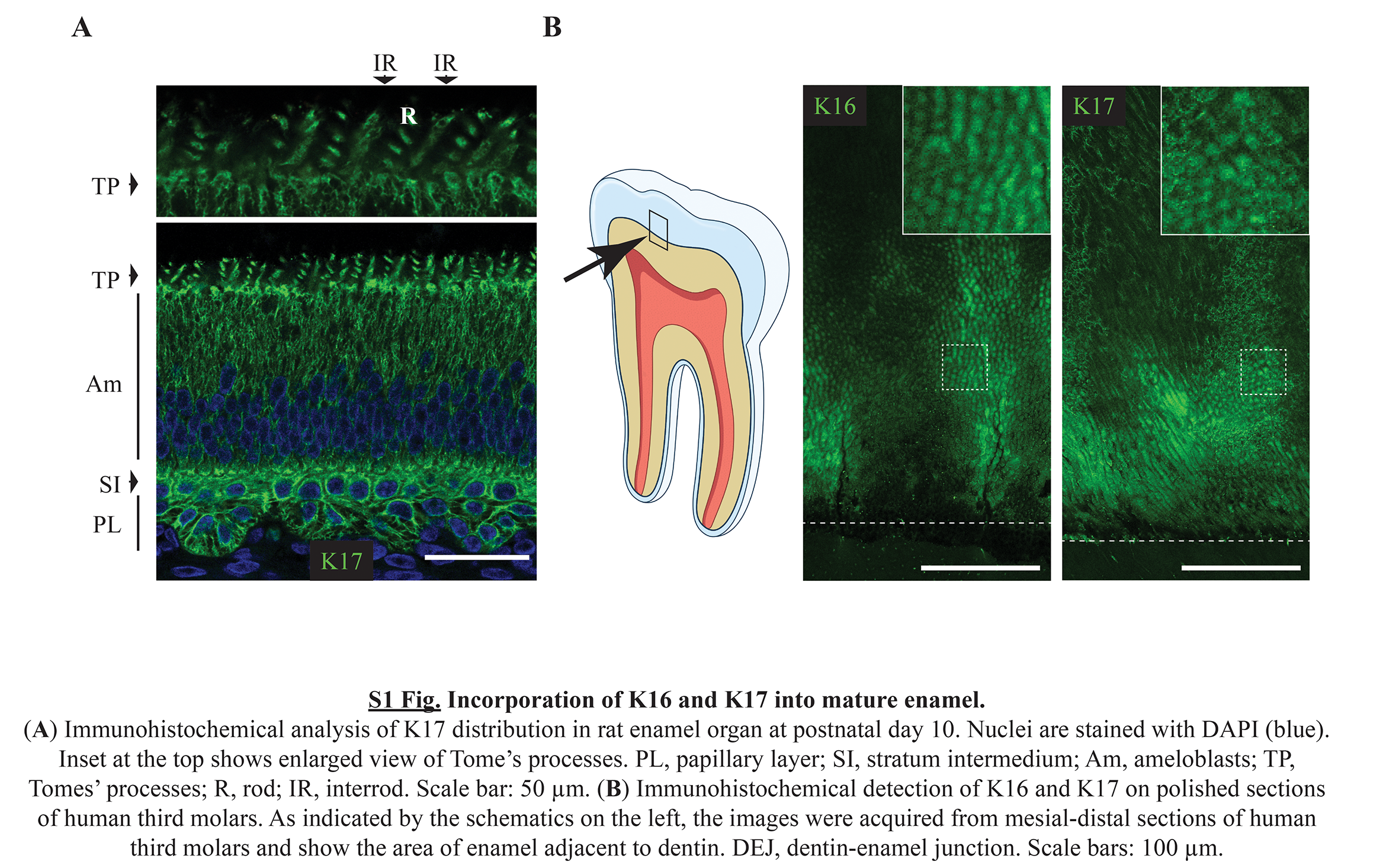

Supplement: S1 Fig — (A) Immunohistochemical analysis of K17 distribution in rat enamel organ at postnatal day 10. Nuclei are stained with DAPI (blue). Inset at the top shows enlarged view of Tomes' processes. PL, papillary layer; SI, stratum intermedium; Am, ameloblasts; TP, Tomes’ processes; R, rod; IR, interrod. Scale bar: 50 μm. (B) Immunohistochemical detection of K16 and K17 on polished sections of human third molars. As indicated by the schematics on the left, the images were acquired from mesial-distal sections of human third molars and show the area of enamel adjacent to dentin. DEJ, dentin-enamel junction. Scale bars: 100 μm. (TIF) [file pgen.1007168.s003.tif]

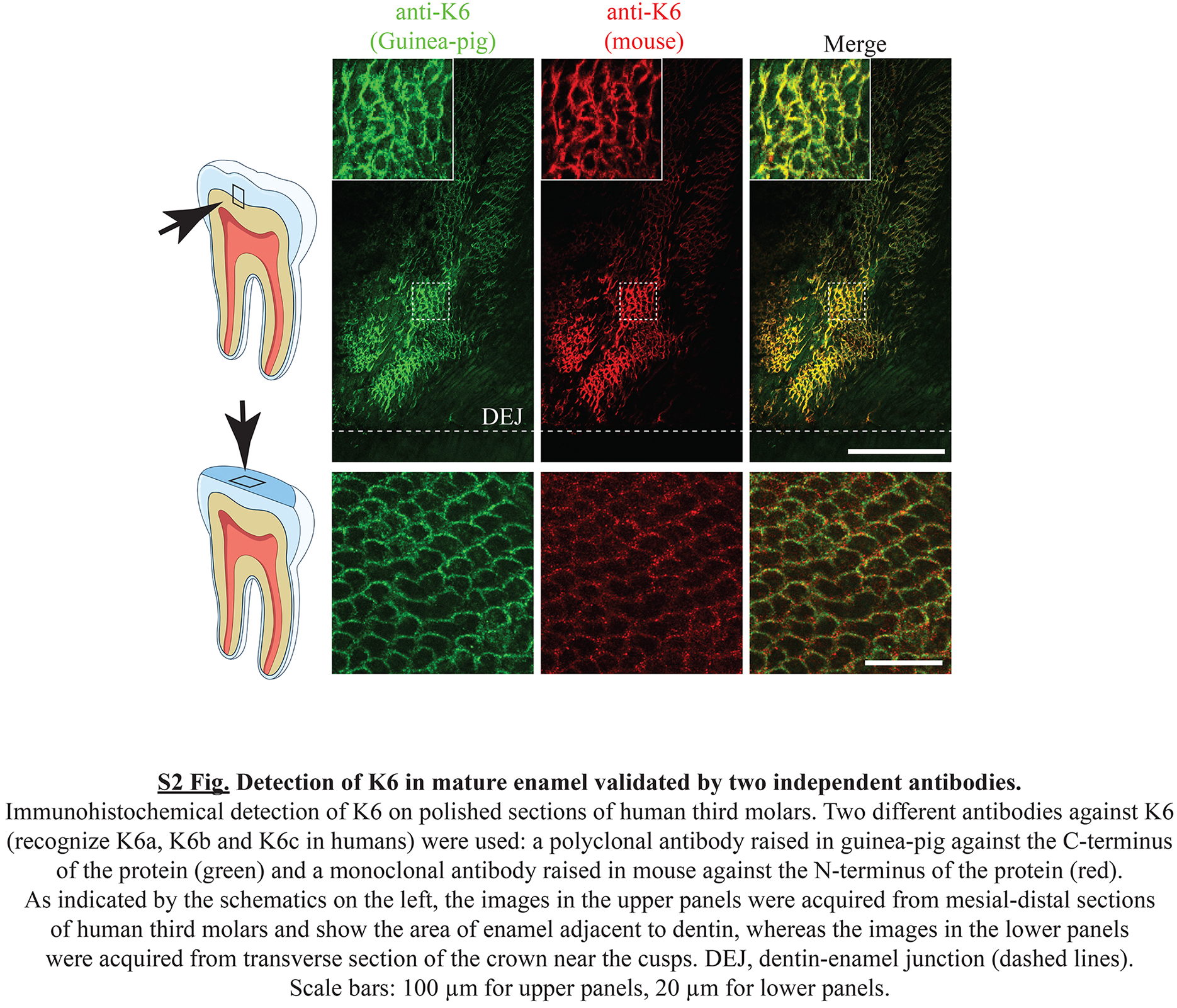

Supplement: S2 Fig — Immunohistochemical detection of K6 on polished sections of human third molars. Two different antibodies against K6 (recognize K6a, K6b and K6c in humans) were used: a polyclonal antibody raised in guinea-pig against the C-terminus of the protein (green) and a monoclonal antibody raised in mouse against the N-terminus of the protein (red). As indicated by the schematics on the left, the images in the upper panels were acquired from mesial-distal sections of human third molars and show the area of enamel adjacent to dentin, whereas the images in the lower panels were acquired from transverse section of the crown near the cusps. DEJ, dentin-enamel junction (dashed lines). Scale bars: 100 μm for upper panels, 20 μm for lower panels. (TIF) [file pgen.1007168.s004.tif]

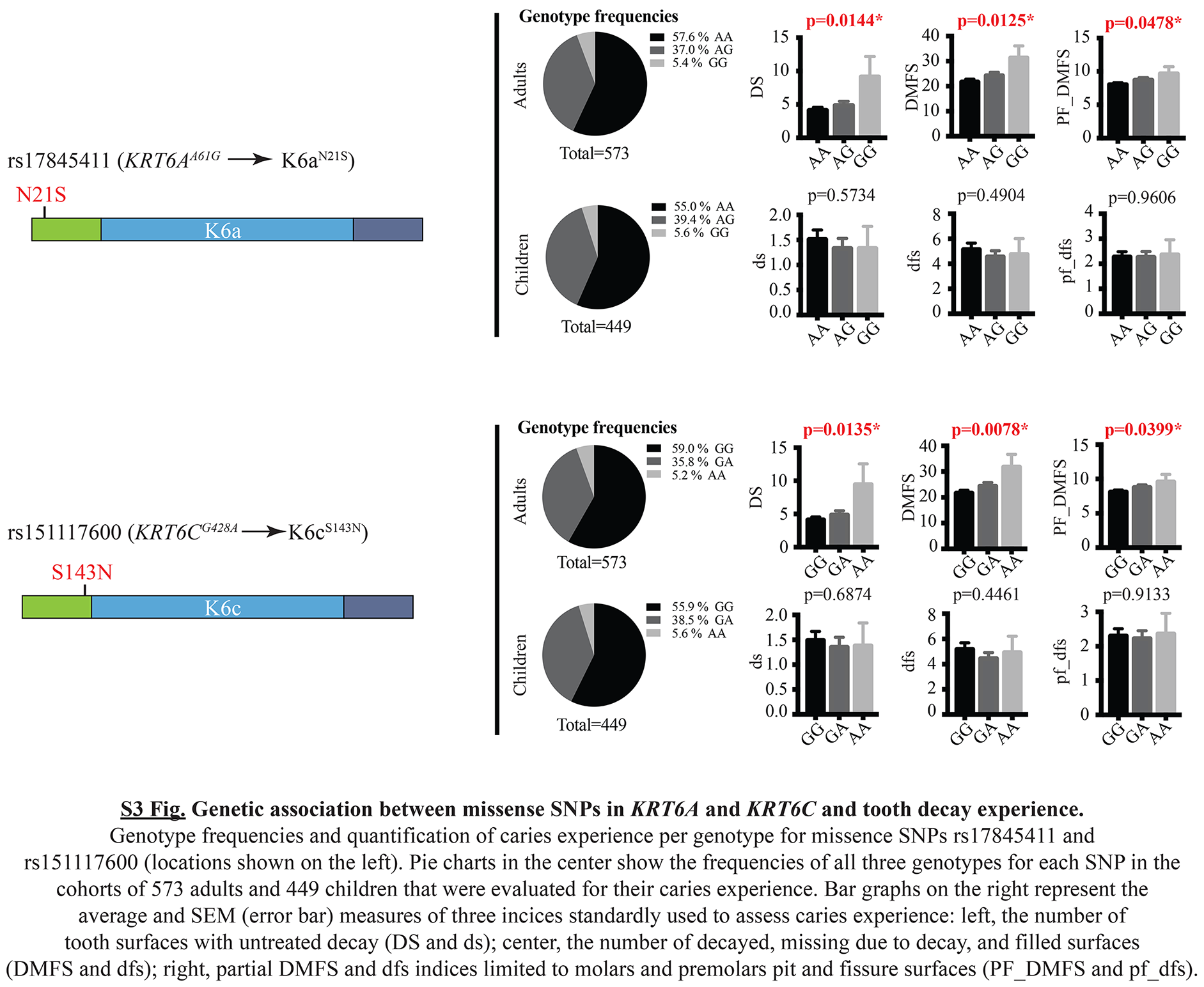

Supplement: S3 Fig — Genotype frequencies and quantification of caries experience per genotype for missence SNPs rs17845411 and rs151117600 (locations shown on the left). Pie charts in the center show the frequencies of all three genotypes for each SNP in the cohorts of 573 adults and 449 children that were evaluated for their caries experience. Bar graphs on the right represent the average and SEM (error bar) measures of three incices standardly used to assess caries experience: left, the number of tooth surfaces with untreated decay (DS and ds); center, the number of decayed, missing due to decay, and filled surfaces (DMFS and dfs); right, partial DMFS and dfs indices limited to molars and premolars pit and fissure surfaces (PF_DMFS and pf_dfs). (TIF) [file pgen.1007168.s005.tif]

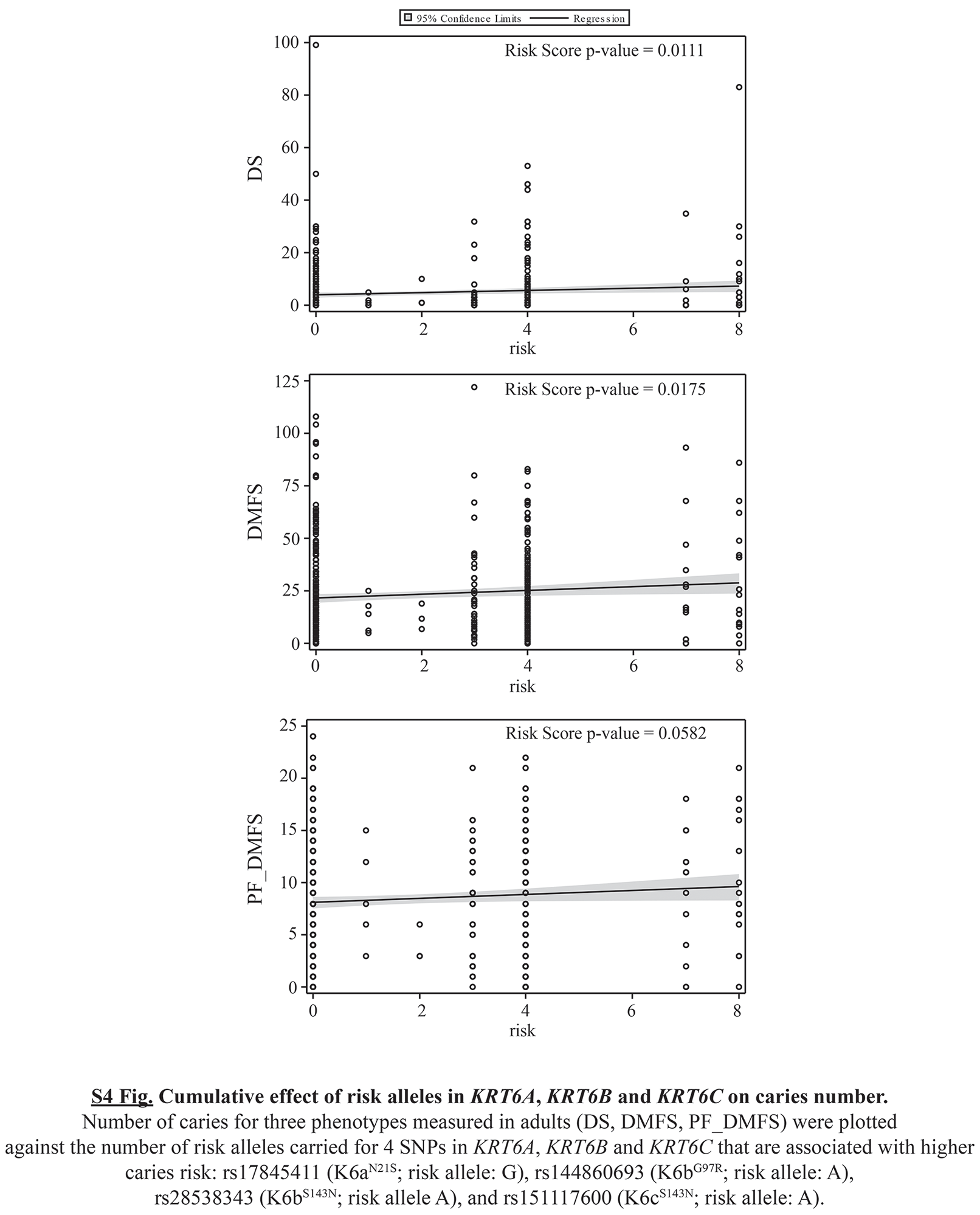

Supplement: S4 Fig — Number of caries for three phenotypes measured in adults (DS, DMFS, PF_DMFS) were plotted against the number of risk alleles carried for 4 SNPs in KRT6A, KRT6B and KRT6C that are associated with higher caries risk: rs17845411 (K6aN21S; risk allele: G), rs144860693 (K6bG97R; risk allele: A), rs28538343 (K6bS143N; risk allele A), and rs151117600 (K6cS143N; risk allele: A). (TIF) [file pgen.1007168.s006.tif]

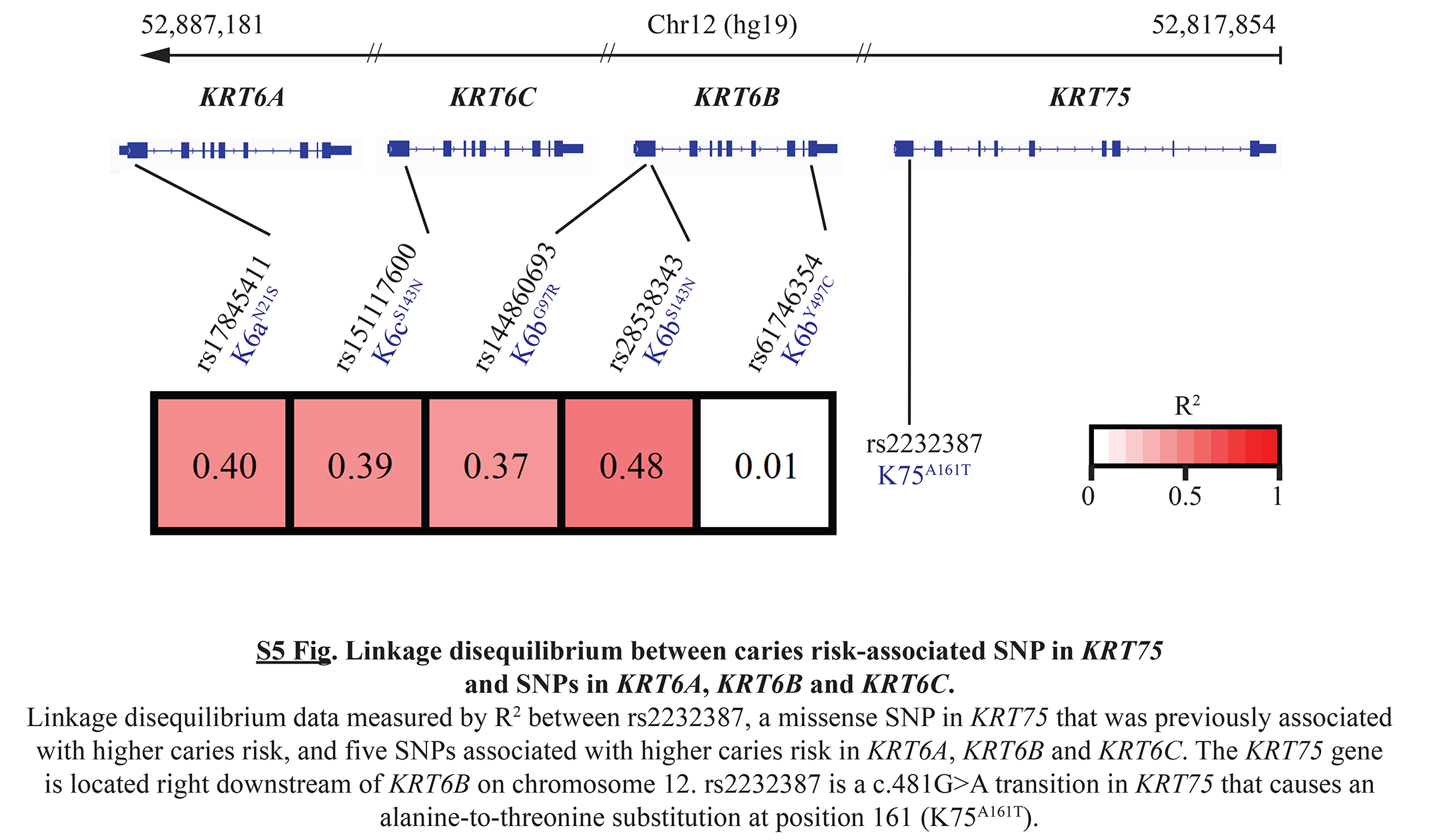

Supplement: S5 Fig — Linkage disequilibrium data measured by R2 between rs2232387, a missense SNP in KRT75 that was previously associated with higher caries risk, and five SNPs associated with higher caries risk in KRT6A, KRT6B and KRT6C. The KRT75 gene is located right downstream of KRT6B on chromosome 12. rs2232387 is a c.481G>A transition in KRT75 that causes an alanine-to-threonine substitution at position 161 (K75A161T). (TIF) [file pgen.1007168.s007.tif]

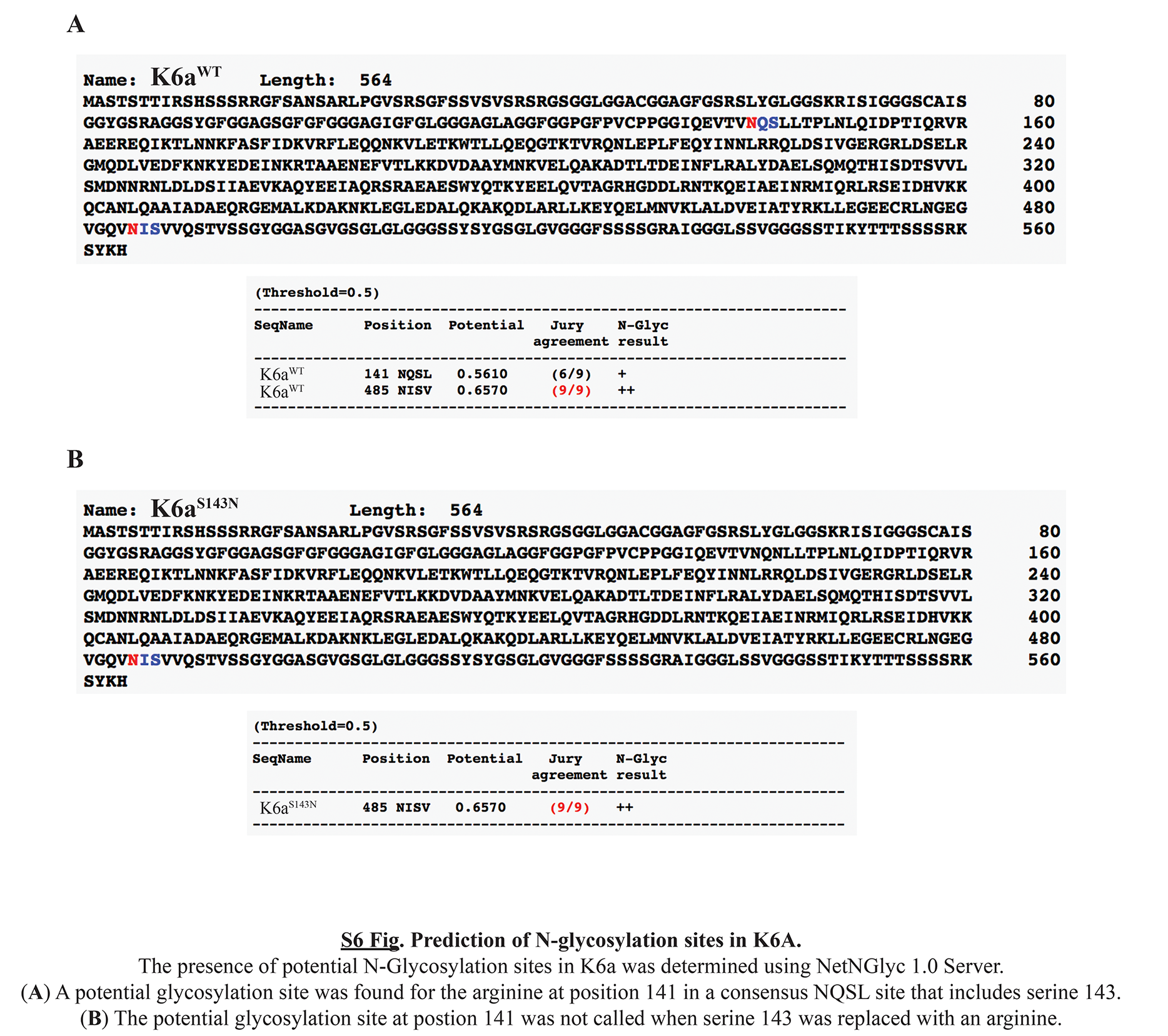

Supplement: S6 Fig — The presence of potential N-Glycosylation sites in K6a was determined using NetNGlyc 1.0 Server. (A) A potential glycosylation site was found for the arginine at position 141 in a consensus NQSL site that includes serine 143. (B) The potential glycosylation site at position 141 was not called when serine 143 was replaced with an arginine. (TIF) [file pgen.1007168.s008.tif]

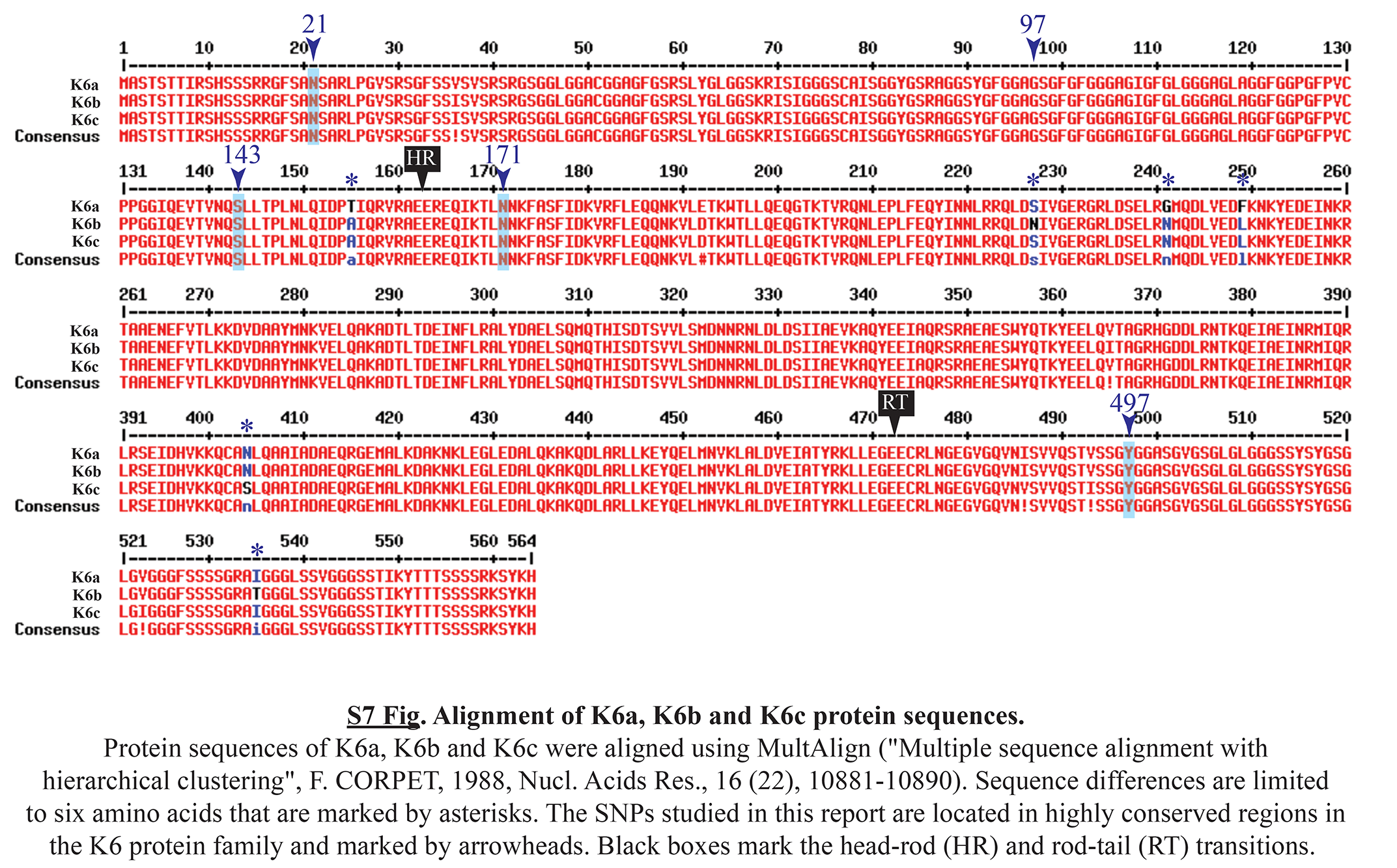

Supplement: S7 Fig — (TIF) [file pgen.1007168.s009.tif]

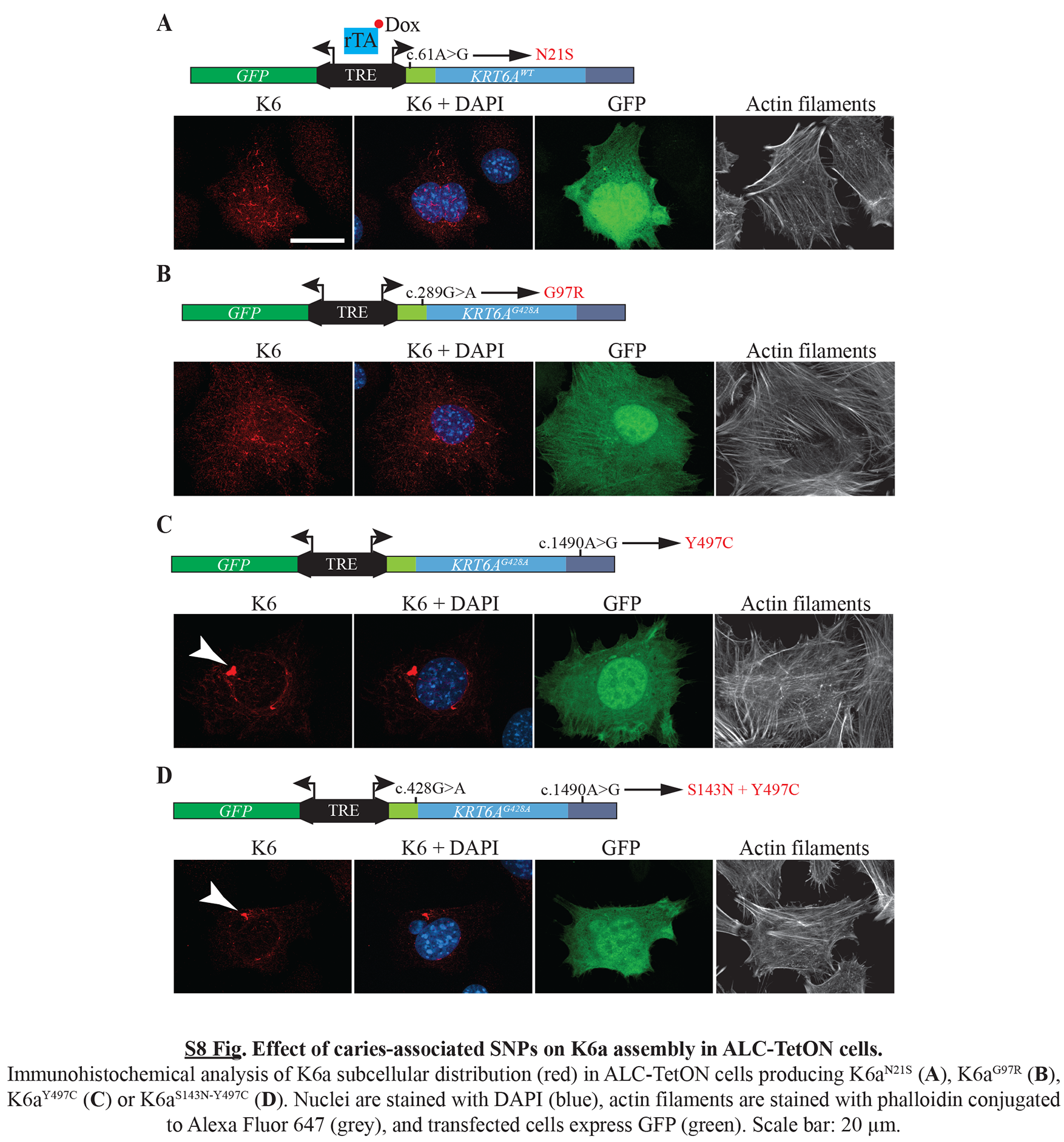

Supplement: S8 Fig — Immunohistochemical analysis of K6a subcellular distribution (red) in ALC-TetON cells producing K6aN21S (A), K6aG97R (B), K6aY497C (C) or K6aS143N-Y497C (D). Nuclei are stained with DAPI (blue), actin filaments are stained with phalloidin conjugated to Alexa Fluor 647 (grey), and transfected cells express GFP (green). Scale bar: 20 μm. (TIF) [file pgen.1007168.s010.tif]
